# A multifunctional integrated biomimetic spore nanoplatform for successively overcoming oral biological barriers

**DOI:** 10.1186/s12951-023-01995-z

**Published:** 2023-08-29

**Authors:** Qingling Song, Junfei Yang, Xiaocui Wu, Yao Li, Hongjuan Zhao, Qianhua Feng, Zhenzhong Zhang, Yun Zhang, Lei Wang

**Affiliations:** 1 School of Pharmaceutical Sciences, Zhengzhou University, Zhengzhou, 450001 People’s Republic of China; 2 Henan Key Laboratory of Targeting Therapy and Diagnosis for Critical Diseases, Zhengzhou, 450001 People’s Republic of China; 3 Key Laboratory of Advanced Drug Preparation Technologies, Ministry of Education, Zhengzhou, 450001 People’s Republic of China

**Keywords:** Biomimetic spore, Oral drug delivery, Spore capsid, Biological barrier

## Abstract

The biological barriers have seriously restricted the efficacious responses of oral delivery system in diseases treatment. Utilizing a carrier based on the single construction means is hard to overcome these obstacles simultaneously because the complex gastrointestinal tract environment requires carrier to have different or even contradictory properties. Interestingly, spore capsid (SC) integrates many unique biological characteristics, such as high resistance, good stability etc. This fact offers a boundless source of inspiration for the construction of multi-functional oral nanoplatform based on SC without further modification. Herein, we develop a type of biomimetic spore nanoplatform (SC@DS NPs) to successively overcome oral biological barriers. Firstly, doxorubicin (DOX) and sorafenib (SOR) are self-assembled to form carrier-free nanoparticles (DS NPs). Subsequently, SC is effectively separated from probiotic spores and served as a functional vehicle for delivering DS NPs. As expect, SC@DS NPs can efficaciously pass through the rugged stomach environment after oral administration and further be transported to the intestine. Surprisingly, we find that SC@DS NPs exhibit a significant improvement in the aspects of mucus penetration and transepithelial transport, which is related to the protein species of SC. This study demonstrates that SC@DS NPs can efficiently overcome multiple biological barriers and improve the therapeutic effect.

**Supplementary Information:**

The online version contains supplementary material available at 10.1186/s12951-023-01995-z.

## Introduction

The gastrointestinal tract (GIT) is the principal region of most oral drugs delivery and therapeutic interventions [1]. Nevertheless, the multiple biological barriers of GIT have seriously restricted the efficacious responses of oral nanoparticles (NPs) in the intestinal disease treatment [2, 3]. Considering the complexity of GIT environment, an optimal oral delivery system should be stable to prevent the premature drug release in the stomach [4]. Moreover, the delivery system also should efficiently address the critical obstacles including mucus and epithelial barriers, which requires the carrier to have different or even contradictory properties [5, 6]. For instance, the NPs surface that is close to electroneutrality charge is beneficial for escaping the mucus entrapment, but unfavorable to the epithelial cellular uptake, resulting in poor absorption and transepithelial transport [7]. At present, some substantial researches have constructed a variety of drug delivery carriers which could overcome these barriers by incorporating some specific functionalities and moieties mechanically [8–10]. However, the synthetic or modified carriers are complex and uncontrollable after administration, which might hinder the further absorption efficiency of NPs. Consequently, development of a simple and stable oral delivery nanoplatform for successively overcoming these aforementioned dilemmas is the focus of current research.

Recently, biomimetic strategy has emerged prevalent across a variety of fields such as drug delivery, biomaterial design and development, which has led to exciting advances [9, 11–14]. Interestingly, the unique protective mechanisms observed in nature, such as plant seed dormancy [15] and bacteria endospores [16], can protect their species from the interference of external environment, providing a blueprint for the development of “biomimetic nanoplatform”. Particularly, as the intrinsic structure of probiotic spores, the spore capsid (SC) integrates many unique biological characteristics, such as high resistance, good stability etc. [17–19]., which can be considered using in the construction of oral biomimetic spore system. However, SC would be disintegrated in the intestine during the spores germination and cannot be effectively utilized as the vehicle for delivering oral drugs [20]. As such, rational capitalizing SC based on a simple and efficient separation method is essential for the development of “biomimetic spore nanoplatform”. In this study, we found that SC containing various protein species which endowed it some unique biological activities such as “muco-inert” property and multi-receptor mediated endocytosis. These promising characteristics would provide clues to find alternative solutions for the oral drugs delivery.

In addition, combinational chemotherapy is still regarded as a primary strategy in the clinical treatment of colon cancer because it can overcome the drug resistance and heterogeneity issue of tumor cells [21–24]. As we previously reported, the chemotherapeutic (doxorubicin hydrochloride, DOX) and molecular targeted drugs (sorafenib, SOR) can be served as a synergistic therapeutic drug to enhance the therapeutic effect of colon cancer [25]. Although this synergistic strategy is promising, how to overcome the inherent limitations of these two free drugs, such as poor water solubility [26], rapid blood clearance [27] and low bioavailability [28], is still a big challenge. Interestingly, DOX with π-conjugated structure and a large number of hydroxyl groups can form stable hydrogen bonds with SOR. Therefore, we reasonably speculated that DOX and SOR could be self-assembled to form carrier-free NPs (DOX/SOR NPs, DS NPs) through the interaction of hydrogen bonds and π-π stacking [29, 30]. Unfortunately, the DS NPs cannot resist to the harsh stomach environment and their use is usually limited by the side-effects that caused by systemic drug exposure.

**Scheme 1:**
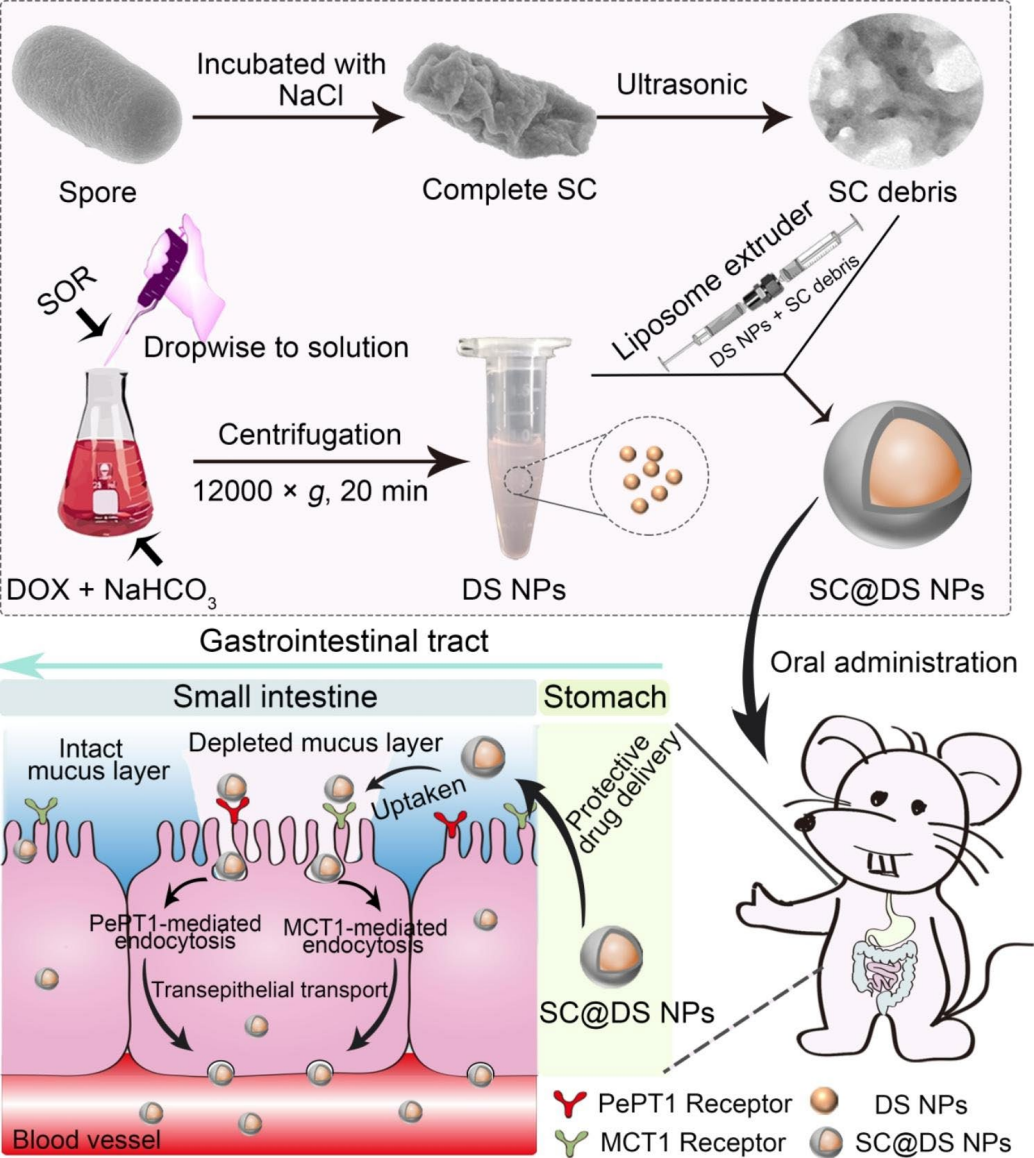
Schematic diagram of fabrication and oral delivery mechanism of the biomimetic spore nanoplatform

Consequently, drawing inspiration by these unique characteristics of SC observed in probiotic spores, we firstly separated the total SC using an original method and determined its protein species by mass spectrometric analysis. And then based on SC, a new type of multifunctional integrated biomimetic spore nanoplatform (SC@DS NPs) was developed to successively overcome these aforementioned concerns in one-stop (Scheme 1). In this system, DOX and SOR were firstly prepared to be DS NPs by self-assembly and subsequently coated with SC that was separated from the probiotic spores. As expected, the results demonstrated that SC@DS NPs can successfully pass through the complex stomach environment, which was attributed to the protective delivery of SC. In addition, SC is rich in cysteine and with a large number of sulfhydryl groups, which can cleave the disulfide bonds between mucus glycoproteins, leading to good effect of evading the trap of mucus. Surprisingly, we found that SC@DS NPs exhibited a significant transepithelial transport efficiency, which was related to the protein species of SC. We demonstrated that such biomimetic spore nanoplatform can successively overcome the mucosal diffusion barrier as well as the epithelial absorption barrier. Our work provided a simple and safe biomimetic nanoplatform that may help to guide the development of oral NPs delivery system.

## Results and discussion

### The preparation and characterization of SC@DS NPs

**Fig. 1:**
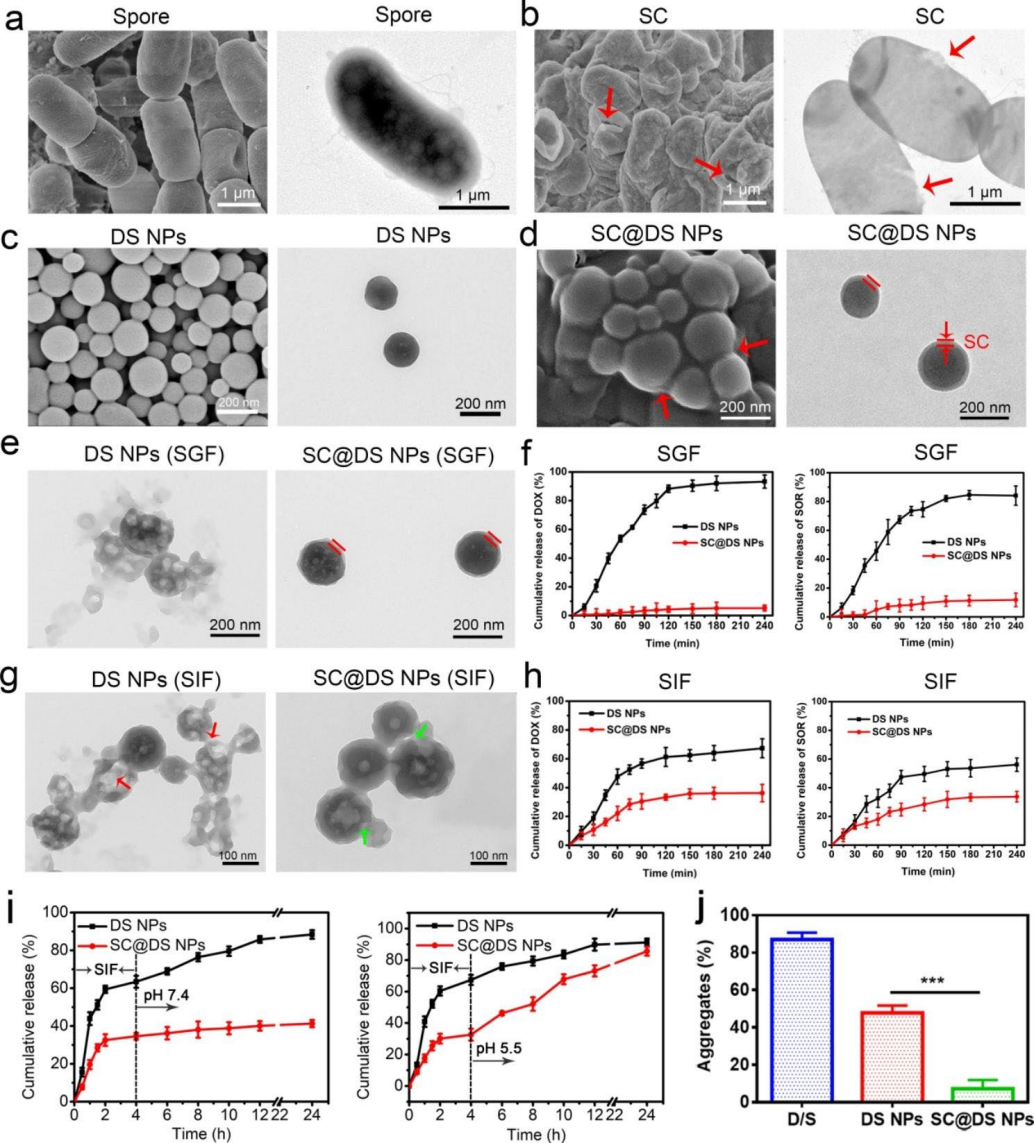
Preparation and characterization. SEM and TEM images of (**a**) spores and (**b**) SC, respectively. The SEM and TEM images of (**c**) DS NPs and (**d**) SC@DS NPs, respectively. (**e**) The morphologies of DS NPs and SC@DS NPs were investigated after 2 h incubation in SGF. (**f**) The cumulative DOX and SOR release of DS NPs and SC@DS NPs in SGF. (**g**) The morphology changes of DS NPs and SC@DS NPs after 4 h incubation in SIF. (**h**) The cumulative release amount of SOR and DOX in DS NPs and SC@DS NPs groups after being incubated in SIF for 4 h. (**i**) The release behavior of drugs from DS NPs and SC@DS NPs after being incubated in a buffer with gradually varying pH for over 24 h. (**j**) The percentage of particles-mucin aggregation formed in simulated mucus solution for different preparations at 37℃

Firstly, the morphology of BC and spore was characterized by Scanning Electron Microscopy (SEM) and Transmission Electron Microscopy (TEM), respectively. As can be seen, BC with the particle size of 3 ~ 5 μm was in rod-shaped (Additional file 1: Fig. S1), while spores of 1 ~ 2 μm with plump and integrity shape were separated successfully from BC (Fig. 1a). Moreover, Gram straining also confirmed that the successful cultivation of BC and isolation of spores (Additional file 1: Fig. S2). Subsequently, in order to prepare spore capsid (SC), BC spores were incubated with different concentration sodium chloride (NaCl) solution. Compared with the smooth surface of spores that dispersed in saline (Fig. 1a), slight fold was observed on the surface of spores after being incubated for 2 h in 5% NaCl (Additional file 1: Fig. S3a), and the shrinkage degree of spores increased with the NaCl concentration increasing (Additional file 1: Fig. S3b). Therefore, in this study, spores were dispersed in 20% NaCl to obtain the pure SC. As shown in Fig. 1b, more severe morphology shrinkage of spores was observed. Moreover, the TEM image showed the clear breaches in the spores where the red arrows point, which could cause the main content was released from spores. This phenomenon indicated that SC was isolated successfully from the spores. Given the high resistance of SC to the harsh environment [17], we speculated that SC could be served as a vehicle for delivering the nanoparticles (NPs) by oral administration. And then SC was broken by Ultrasonic Homogenizer to prepare SC debris. As shown in the Additional file 1: Fig. S4, BC presented lamellar structure, which was conducive to the preparation and formation of NPs and drugs loading.

Furthermore, the strategy of combination of two drugs can be used to enhance the therapeutic effect [31–33]. Accordingly, as a proof of concept, the water-soluble DOX and hydrophobic SOR were selected to form DS NPs by the nanoprecipitation method [30]. Surprisingly, as shown in Fig. 1c and Additional file 1: Fig. S5, the DS NPs were of good morphology and well dispersion. The UV absorption peak at 266 nm (red arrow) and 480 nm (black arrow) of the DS NPs was consistent with the maximum absorption peak of free SOR and DOX, respectively, which further proved that the composition of DS NPs was DOX and SOR (Additional file 1: Fig. S6). Subsequently, as shown in Fig. 1d and Additional file 1: Table S1, compared with DS NPs, the thick coating was observed on the surface of SC@DS NPs, which confirmed that SC@DS NPs were synthesized successfully after being extruded for 13 times. Moreover, the potent wrapping of SC onto the surface of DS NPs was further demonstrated by the slight particle size increasing and surface charge variation compared with the uncoated DS NPs (Additional file 1: Fig. S7 and S8). Notably, the protein composition of SC@DS NPs was similar to that of SC, indicating that SC coating process could hardly affect the surface composition of SC (Additional file 1: Fig. S9). With extension of the storage time at room temperature, the negligible changes of particle size and zeta potential illustrated the superior stability of SC@DS NPs (Additional file 1: Fig. S10 and S11). Additionally, the stability of SC@DS NPs in PBS, serum and cell culture media also was evaluated. As shown in Additional file 1: Fig. S12, SC@DS NPs were in a good dispersion in various biological media, indicating their good biocompatibility and biological stability.

Considering that spore coat is the key factor of spore resistance to the harsh acidic environment, we speculated that SC was relatively stable in stomach. In order to study whether SC could protect DS NPs from the extreme stomach condition, the DS NPs and SC@DS NPs were incubated in the simulated gastric fluid (SGF), respectively. After 2 h incubation, their morphologies were observed by TEM images. As shown in Fig. 1e, DS NPs were destroyed while SC@DS NPs could maintain the complete shape, which suggested that SC could be served as the protective vehicle. In addition, during the incubation in SGF, the drug release of DOX and SOR was also evaluated. As can be seen in Fig. 1f, the release amounts of DOX and SOR in DS NPs were close to 100%. On the contrary, the negligible release amount was detected in SC@DS NPs, which further testified the protective function of SC. Furthermore, the drug release properties and the morphology changes of DS NPs and SC@DS NPs were evaluated after both of the NPs were incubated in stimulated intestinal fluids (SIF) for 4 h, respectively. As shown in Fig. 1g, most DS NPs were broken (red arrow) and could not maintain a complete morphology. Although the SC@DS NPs could keep a good shape, there were still some slight damages because of the presence of intestinal specific enzymes, which could lead to a low drug release rate of about 30% in the SIF (Fig. 1h). And then, after the DS NPs and SC@DS NPs were pre-incubated in SIF for 4 h, they were transferred to the solution of pH 7.4 and pH 5.5 for another incubation time. As shown in Fig. 1i, the high release rate of DS NPs was detected in both pH 7.4 and pH 5.5. However, for the SC@DS NPs, there was almost no drug release after being incubated in pH 7.4 for 20 h, while the sustained drug release was observed when they were transferred to the pH 5.5 solution. This result suggested that SC@DS NPs could achieve a sustained drug release in the tumor microenvironment.

Notably, mucus barrier could decrease the efficiency of the intestinal epithelial absorption of NPs [34]. We further analyzed the specific protein components of SC by LCMSMS (nanoLC-QE). As shown in Additional file 1: Table S2, the SC contains amount of cysteine, which could lead to the high mucus penetration of SC. As we all know, mucus is composed of the complex biochemical compositions, which could adsorb a wide range of molecules and particles, including drugs, and other potentially harmful entities [35, 36]. As shown in Fig. 1j, a high aggregates rate was detected in the D/S treatment group, which was because the small molecule drugs could be trapped and quickly removed by intestinal mucus. Among these groups, SC@DS NPs showed a low aggregate rate with a percentage of 7.2% ± 4.7%, which could be interpreted as that SC containing amount of cysteine could cleave the disulfide bonds between mucus glycoproteins, increasing the intestinal mucus penetration of DS NPs [37, 38]. Furthermore, the vertical distribution of NPs (red) on cell monolayer were observed by Confocal Laser Scanning Microscopy (CLSM) using Z-axial scanning (Additional file 1: Fig. S13). The fluorescence signals of DOX and DS NPs treatment groups exhibited a strong correlation with the mucus layer while very few signals were overlaid with the mucus for SC@DS NPs, indicating that a large proportion of SC@DS NPs could escape the trapping of mucus. Additionally, we next investigated whether the differences are attributed to alterations in trap or permeability of NPs at the mucosal surface. The significant separation of red and green fluorescence was observed in the SC@DS NPs, indicating its superior ability to escape from mucus trapping and good permeability (Additional file 1: Fig. S14).

### Uptake mechanism and transepithelial transport of SC@DS NPs

**Fig. 2:**
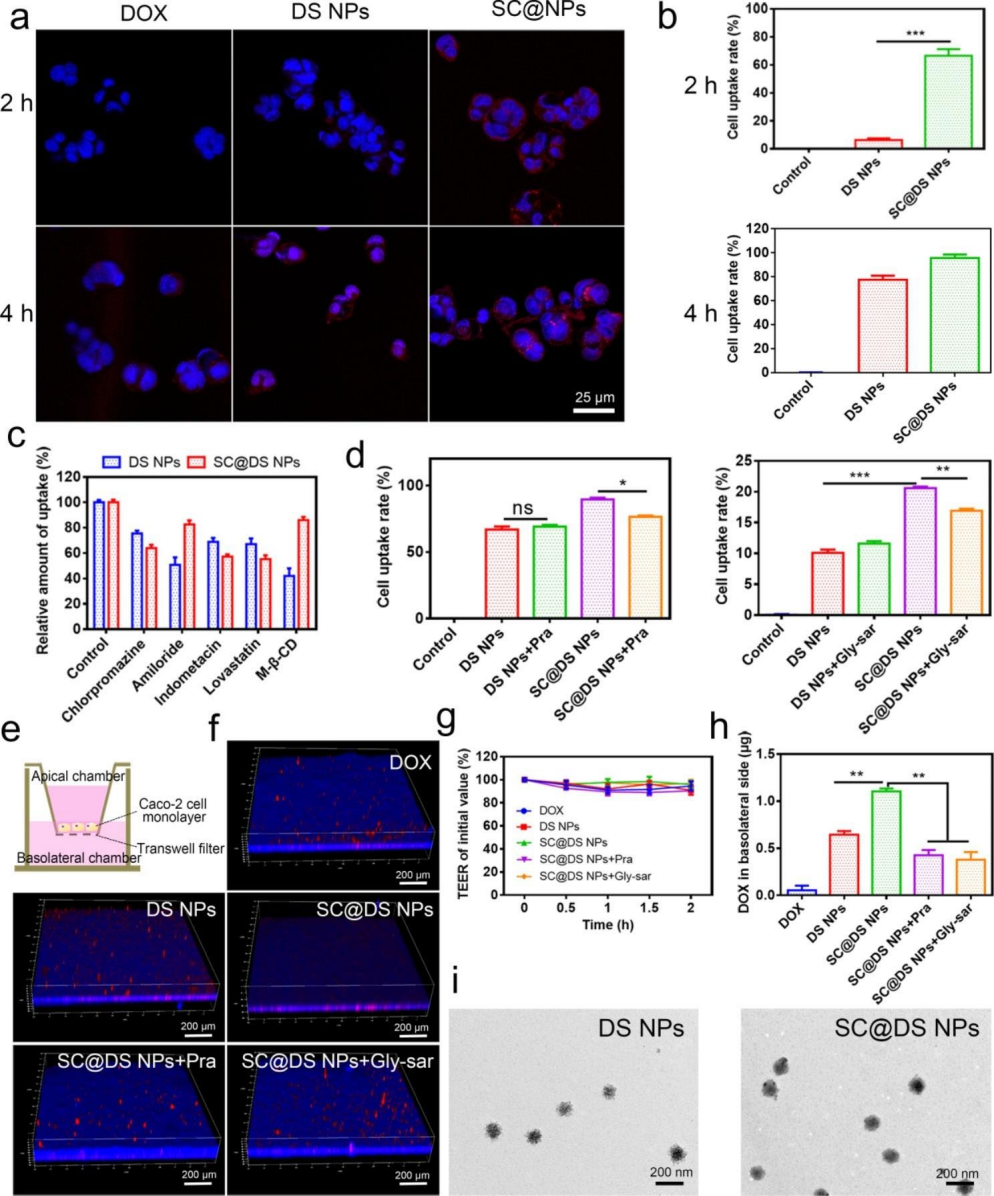
Studies of uptake mechanism and transepithelial transport. (**a**) Cell uptake of free DOX, DS NPs and SC@DS NPs at 2 and 4 h incubation, respectively. (**b**) The result of flow cytometry statistical analysis after the Caco-2 cells being incubated with DS NPs and SC@DS NPs after 2 and 4 h incubation, respectively. (**c**) The measurement of relative uptake on Caco-2 cells after being incubated with the different endocytosis inhibitors. (**d**) The influences of Pra and Gly-sar on the cellular uptake of DS NPs and SC@DS NPs, respectively. (**e**) Establishment of Caco-2 cell monolayer models. (**f**) The CLSM images of different NPs on Caco-2 cell monolayers from apical to basolateral side. (**g**) TEER values of Caco-2 cell monolayers before and after different incubations. (**h**) The amount of transepithelial transport of different groups in the basolateral chamber. (**i**) The TEM images of DS NPs and SC@DS NPs in the basolateral chamber

Efficient epithelium uptake and transepithelial transport play an essential role to the oral NPs delivery [39]. Firstly, the cytotoxicity of SC and SC@DS NPs was evaluated on Caco-2 cells, respectively. When the concentrations of SC and SC@DS NPs reached 500 µg/mL, the cell viability rate was about 95%, indicating that the drug carrier system has good biocompatibility and no obvious toxicity on Caco-2 cells (Additional file 1: Fig. S15). Notably, we found that the cell uptake rate on Caco-2 cells was significantly improved by SC@NPs compared with the other treated groups (Fig. 2a, b and Additional file 1: Fig. S16). This phenomenon is because SC contains some target proteins which could bind to the receptors in epithelium. Intrigued by this promising result, we further investigated the endocytosis mechanism of SC@DS NPs. As shown in Fig. 2c, amiloride and M-β-CD were served as the non-specific inhibitors for macropinocytosis and pathway inhibitor of disruptor of lipid raft, respectively, which could significantly reduce the cellular uptake of the DS NPs treated group. Moreover, chlorpromazine and lovastatin were served as the inhibitors of clathrin-mediated endocytosis, and caveolae-mediated endocytosis pathways, respectively. Interestingly, SC@DS NPs displayed a marked inhibition effect of cellular uptake after being treated with chlorpromazine (11% reduction) and lovastatin (11.9% reduction), respectively. These results indicated that the cellular uptaken efficiency of SC@DS NPs was involved in the multiple cellular pathways such as lipid raft and caveolae-mediated uptake, clathrin-dependent endocytosis as well as macropinocytosis. As expected, this non-specificity of endocytosis pathway could contribute to the improvement of their cellular uptake efficiency.

Furthermore, we also studied the specific receptor-mediated endocytosis that related to the high uptake of SC@DS NPs. The previously studies claimed that the monocarboxylate transporter-1 (MCT1) was the main receptor in the transport of short-chain fatty acid (SCFA), such as butyrate, lactate and propionate [40]. As shown in Fig. 2d, the cellular uptake of SC@DS NPs was evidently depressed by pravastatin (Pra) known as the inhibitor of MCT1. This result positively supported that MCT1 played a vital role for the endocytosis of SCFA-functionalized nanovehicles. Besides, the increased uptake rate in SC@DS NPs group might be associated with the extra activation of the other pathways. As reported, SC contains amount of tyrosine, which might enhanced the receptor-mediated endocytosis by the oligopeptide transporter (PePT1) [41]. The glycyl-sarcosine (Gly-Sar), as the inhibitor of the PePT1 receptor, could significantly decrease the uptake of SC@DS NPs. Without specific interaction with MCT1, SC@DS NPs still showed the high cellular uptake rate.

Subsequently, the in vitro transepithelial transport of different NPs was evaluated by the Caco-2 cell monolayers (Fig. 2e). To our surprise, as shown in Fig. 2f, the fluorescence of SC@DS NPs treatment group was embedded in the cell monolayer penetrating to the basal side of the monolayer. While the fluorescence of other groups was floating on the monolayer, and the penetrating phenomenon of these NPs was hardly observed. However, SC@DS NPs hardly achieved efficient transepithelial transport when the cell monolayers were incubated with the different pathway inhibitors, respectively. This result was interpreted by the fact that the transport efficiency of SC@DS NPs could be enhanced by the receptor mediated endocytosis. Additionally, the transepithelial electrical resistance (TEER) values of cell monolayer were nearly unchanged before and after the different treatments (Fig. 2g). And the tight junction protein was presented at the border of epithelial cells and the apical cellular membrane, and the positive expression of Occludin in sections is marked red color (Additional file 1: Fig. S17). Interestingly, consistent with the control group, the annular and continuous red fluorescence was observed around the nucleus in the SC@DS NPs group. However, the discontinuous red fluorescence was detected in the other treated groups. These results all proved that the permeability of SC@DS NPs was caused by receptor-mediated transcellular transport rather than breakage of the integrity of cell monolayers. The amount of transcellular transport of SC@DS NPs was measured in basolateral chamber, exhibiting 2.6-fold and 2.9-fold (*P* < 0.05) higher than that of the group pre-treatment with Pra and Gly-sar, respectively (Fig. 2h). Meanwhile, the morphology of different NPs in basolateral chamber was detected by TEM. As shown in Fig. 2i, the edge of DS NPs was rough while the surface of SC@DS NPs with integrity was smooth, which indicated that SC@DS NPs had good stability when they were transported across the epithelium.

### Evaluation of anti-tumor efficiency in vitro

**Fig. 3:**
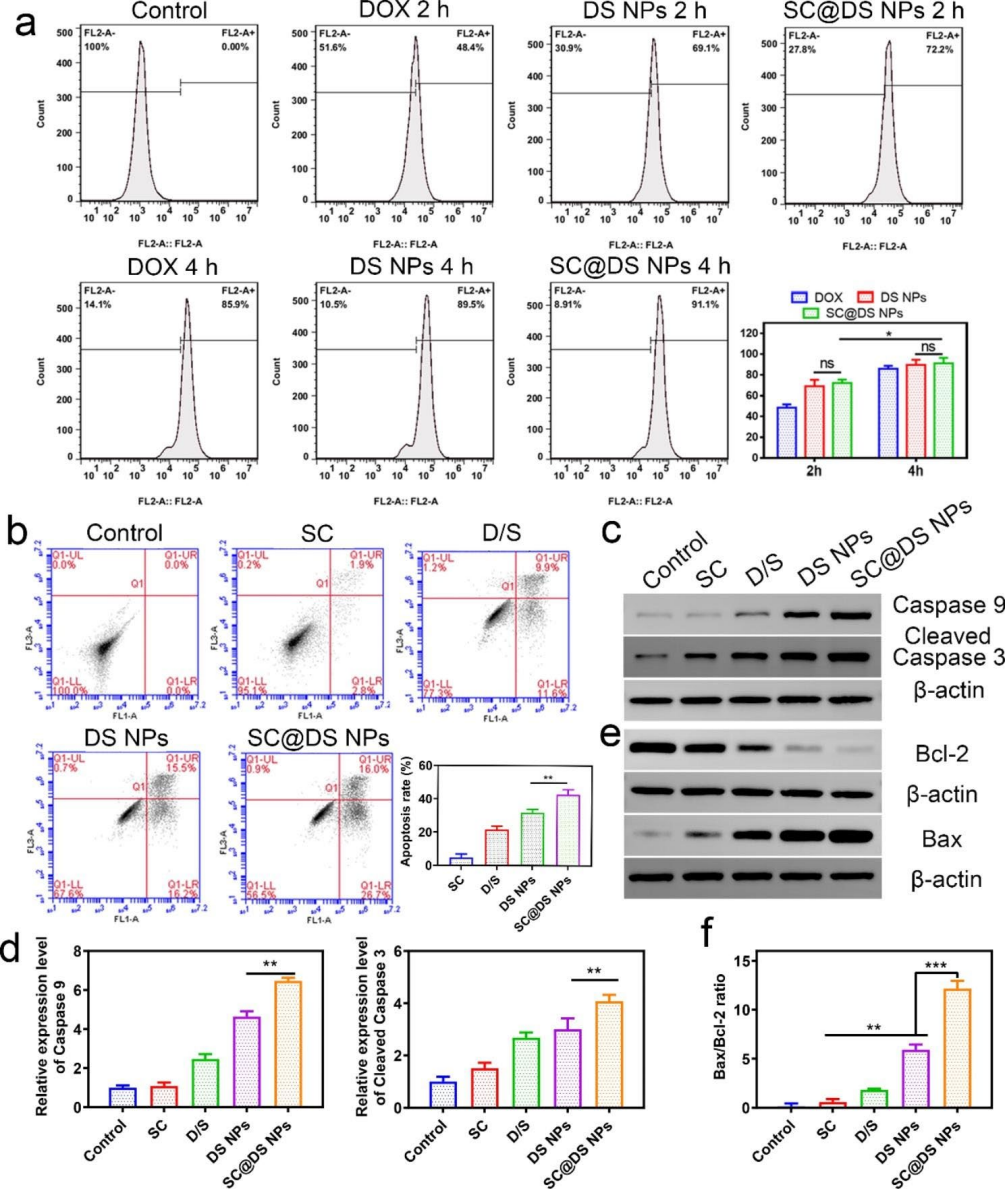
Cellular uptake and apoptosis analysis in SW620 tumor cells. (**a**) Quantitative determination of cell uptake amounts for free DOX, DS NPs and SC@DS NPs at 2 and 4 h incubation, respectively. (**b**) Apoptosis level of SW620 cells after being treated with D/S, DS NPs, SC and SC@DS NPs, respectively. (**c**) The caspase-9 and cleaved-caspase-3 protein levels of SW620 cells and (**d**) semi-quantitative analysis of different groups. (**e**) The bax/bcl-2 ratio analysis

The in vitro anti-tumor efficiency was investigated by the human colon tumor cell line SW620. As shown in Fig. 3a and Additional file 1: Fig. S18, there were no significant differences between DS NPs and SC@DS NPs treatment groups after 2 and 4 h incubation, respectively. This result suggested that SC could only improve the intestinal epithelial transport efficiency due to the specific receptors, and it could not perform obvious cellular uptake in tumor cells. However, the cell uptake of DS NPs and SC@DS NPs was observably higher than that of DOX with the incubation time. Besides, the apoptosis level of different group in SW620 cells was also detected. As shown in Fig. 3b, there were more apoptotic cells in SC@DS NPs group with an apoptotic rate of 42.7 ± 3.0%, which was significantly higher than that of DS NPs treatment group (21.7 ± 2.2%) and D/S treatment group (21.5 ± 2.1%). This result was attributed to the fact that SC also could induce a mild apoptosis on tumor cells. The apoptosis level was further determined by Western blotting analysis. As shown in Fig. 3c, d and Additional file 1: Fig. S19, a significant improvement in the apoptosis proteins such as caspase-9 and cleaved caspase-3 was observed after incubation with SC@DS NPs, indicating that SC@DS NPs could induce severe cell apoptosis. Moreover, the level of tumor cell apoptosis is also regulated by the antiapoptotic protein bcl-2 and the apoptotic protein bax [42]. The expression of bcl-2 and bax and the semi-quantitative analysis of bcl-2/bax ratio for each group were shown in Fig. 3e, f and Additional file 1: Fig. S20. For the SC@DS NPs treatment group, the bax/bcl-2 ratio was remarkably lower than that of the other groups, indicating that this preparation could enhance the process of cell apoptosis.

### Intestinal absorption in vivo

**Fig. 4:**
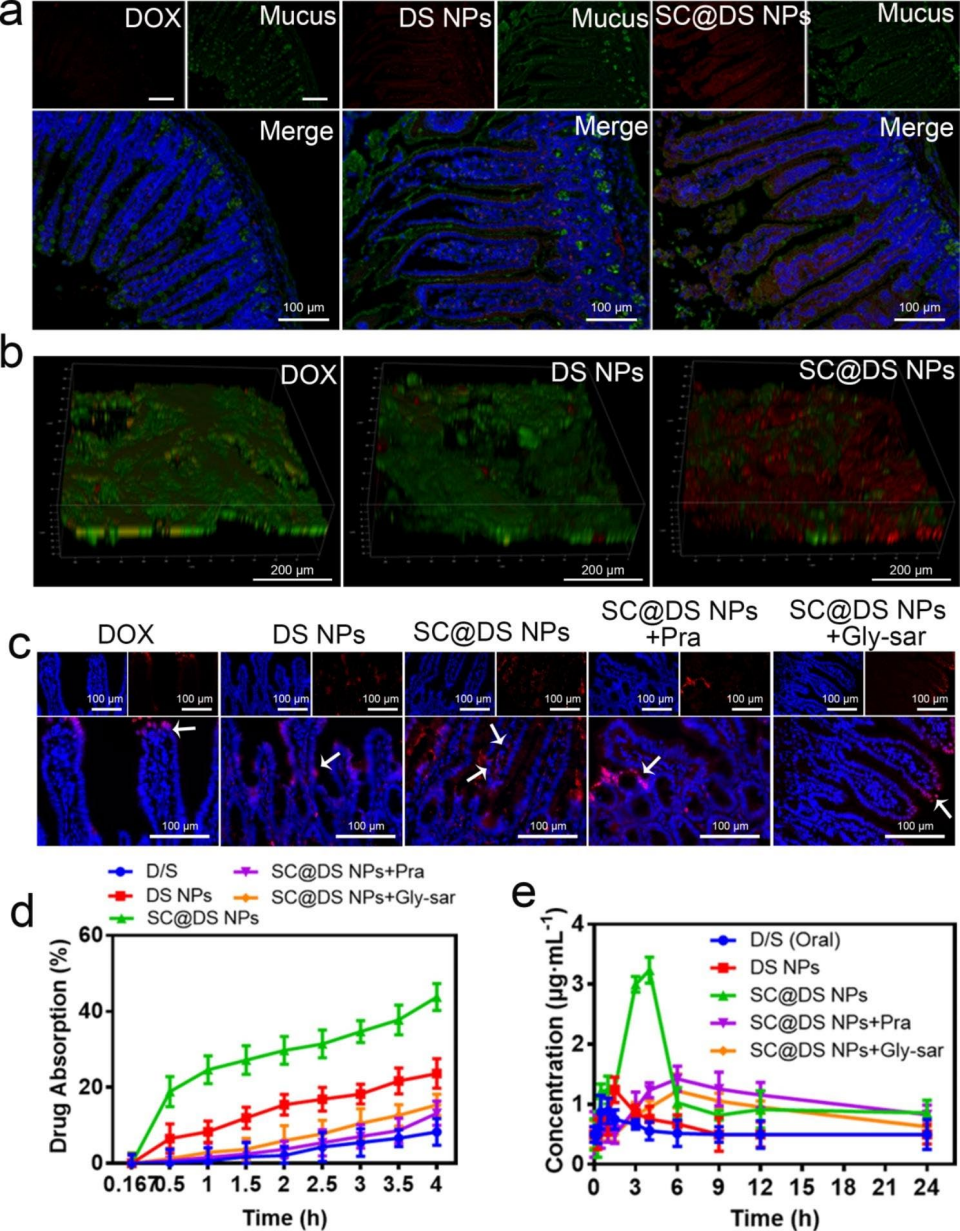
Evaluation of intestinal absorption and pharmacokinetic parameters. (**a**) The mucus penetration effect of different NPs. (**b**) The typical 3D CLSM images of the different NPs distribution in mucus. (**c**) Representative fluorescence images of intestine after the mice were treated with free DOX, DS NPs, SC@DS NPs, SC@DS NPs + Pra and SC@DS NPs + Gly-sar by gavage, respectively. (**d**) Evaluation of the intestinal absorption of different groups by the in situ intestinal circulation and absorption model. (**e**) Drug concentration in plasma after oral administration with different groups at a dose equivalent to 30 mg kg^− 1^ of DOX.

The previous study claimed that the sulfhydryl groups of cysteine on the surface of spores could cleave the disulfide bond between mucin glycoproteins and improve the intestinal mucus penetration of NPs [16]. As shown in Fig. 4a and Additional Fig. S21, low fluorescence intensity was observed in the DOX-treated group, which indicated that DOX could not penetrate the mucus. For the DS NPs-treated group, though the marked fluorescence signal was detected, the intestinal epithelial cellular uptake exhibited a strong correlation with the mucus layer, indicating that a larger proportion of DS NPs was trapped within the mucus. This result was also demonstrated in the in vitro particles-mucin aggregation experiment. On the contrary, the higher fluorescence intensity was noticeably observed in the intestinal villi, while very few fluorescence signals were overlaid with the mucus. Moreover, as shown in Fig. 4b, the 3D images of the intestinal tissues with different treatment also proved that SC@DS NPs could dramatically avoid mucus trapping and improve the uptake efficiency of intestinal epithelial cells. This phenomenon can be interpreted as the protective functional of the SC.

For further investigating the in vivo intestinal absorption of different NPs, the mice were orally treated with free DOX, DS NPs and SC@DS NPs for 4 h, respectively. As shown in Fig. 4c, the weak red fluorescence signal was detected in the intestinal villi after the mice were treated with DS NPs, indicating the poor absorption of DS NPs. As expected, the strong fluorescence signal was obviously observed at both the epithelial cells and basolateral side of the epithelium in the SC@DS NPs treatment group. Interestingly, when the SC@DS NPs treated group was pre-incubated with Pra and Gly-sar, respectively, they exhibited a weak fluorescence signal in the epithelial cells. This result demonstrated that SC@DS NPs were efficiently absorbed at the epithelium and successfully transported into the lamina propria by the receptor-mediated endocytosis. Subsequently, the evaluation of in situ intestinal absorption and circulation was performed by the ex vivo ligated intestinal loop model. As shown in Fig. 4d, the amount of drug absorption of SC@DS NPs was 43.8% ± 3.5%, which was significantly higher than that of DS NPs with the drug absorption of 23.6% ± 3.9%. However, when the transcellular pathway was inhibited by Pra or Gly-sar, the intestinal epithelial absorption of SC@DS NPs was 13.2% ± 3.1% and 15.4% ± 2.8%, respectively. These results suggested that SC@DS NPs could increase the efficiency of transepithelial transport and absorption, which was the outstanding prerequisite to enhance the therapeutic effect.

Given the promising characteristics of SC@DS NPs, we further evaluated the oral relative bioavailability after administration with D/S, DS NPs, SC@DS NPs, SC@DS NPs + Pra and SC@DS NPs + Gly-sar, respectively. The plasma concentration time curves and pharmacokinetic parameters were shown in Fig. 4e and Additional file 1: Table S3. The C_max_ value of DS NPs was 1.24 ± 0.35 µg mL^− 1^ at 1.5 h, which was lower than that of the other treatment groups. This result was mainly due to the poor stability of DS NPs when they passed through the harsh stomach conditions. Notably, the SC@DS NPs treated group achieved the highest relative bioavailability (F_rel_), which was 5.6-fold higher than the values obtained by the DS NPs treatment. In addition, when the mice were pre-treated with the receptor inhibitors and followed by.

treatment with SC@DS NPs, their relative bioavailability was remarkably decreased. Taken together, SC@DS NPs with the superior mucus-penetrating and cellular uptake capabilities could be served as a valuable drug delivery system, improving the drugs bioavailability.

### In vivo distribution and anti-tumor efficacy

**Fig. 5:**
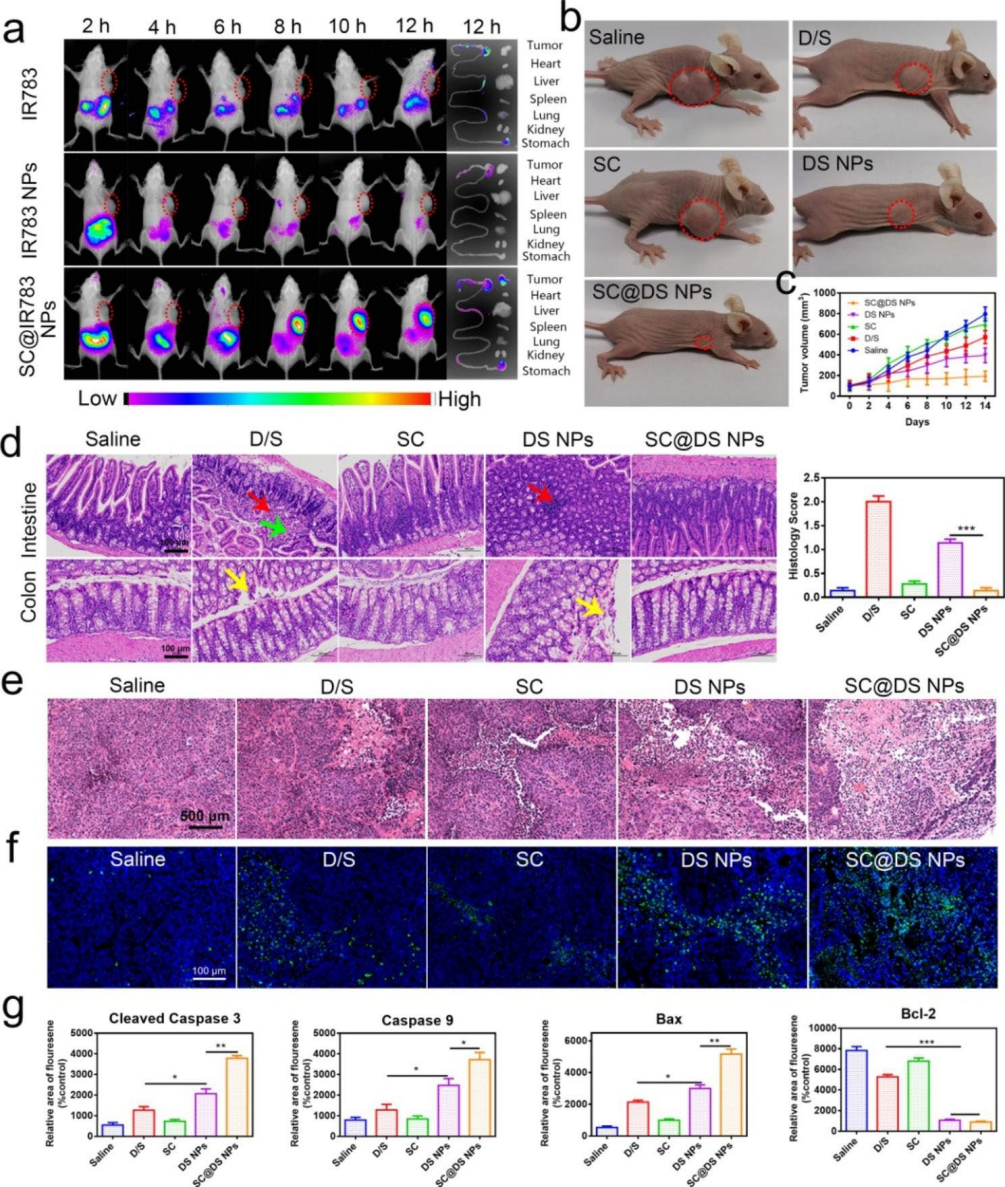
The in vivo distribution and treatment efficacy of different NPs. (**a**) In vivo imaging of SW620 tumor-bearing mice at the preset time points. (**b**) Representative pictures of mice after different treatments. (**c**) Changes in tumor volume during treatment. (**d**) Representative H&E images of intestinal and colorectal tissues and histology score after different treatments (Red arrow: focal lymphocytic infiltration; Yellow arrow: desquamation of epithelial cells, Green arrow: visible connective tissue hyperplasia in the lamina propria); Scale bar = 100 μm. (**e**) H&E staining of tumor tissues from different treatments (Scale bar = 500 μm). (**f**) TUNEL staining of tumor tissues at the end of each treatments (Scale bar = 100 μm). (**g**) Quantitative analysis of apoptosis and anti-apoptosis related proteins including cleaved caspase-3, caspase-9, bax, and bcl-2 in different treatments (mean ± SD, n = 6, *P < 0.05, **P < 0.01, ***P < 0.001). The different formulations were orally administered to rats at a dose equivalent to 30 mg kg^− 1^ of DOX and 5 mg kg^− 1^ of SOR, respectively. Significant differences were assessed using two-way analysis of variance (ANOVA) with multiple comparisons or using the t-test for comparison of two groups

For investigating the in vivo distribution of NPs, a near-infrared dye IR783 was employed to replace DOX to form the IR783 NPs self-assembly. As shown in Fig. 5a, the fluorescence signal notably accumulated into the stomach at 2 h for the free IR783 and IR783 NPs treatments, respectively. As time continued to extend, the rapid decrease of fluorescence distribution in vivo was clearly observed. By contrast, after the mice were treated with SC@IR783 NPs, the tumor site exhibited a strong fluorescence signal at 8 h and remained obvious fluorescence until 12 h, while the other treated groups displayed almost negligible fluorescence signal. This phenomenon of the NPs real-time distribution of the major organs and intestinal tissues could be interpreted as follows: (i) SC could protect NPs from the harsh gastrointestinal tract environment; (ii) SC could efficiently enhance the transepithelial transport ability of NPs by the multiple transport pathways, which could increase the amount of NPs that entered the bloodstream. These promising in vivo distribution characteristics of NPs might lead to a superior anti-tumor efficacy.

Subsequently, the in *vivo* tumor inhibition efficiency was investigated after treatment with saline, D/S, SC, DS NPs and SC@DS NPs for 14 days, respectively. As shown in Additional file 1: Fig. S22, the body weight gain of D/S and SC treated groups was slow because DOX had cardiotoxicity, while SC had only weak therapeutic effect. The body weight of the mice treated with SC@DS NPs was increased obviously, which suggested the good biocompatibility and treatment effect of SC@DS NPs. Moreover, the tumor volumes of each mouse were recorded to evaluate the treatment efficacy of different groups. As depicted in Fig. 5b, c and Additional file 1: Fig. S23, no obvious tumor inhibition was observed in saline and SC treatment groups. By comparing with the tumor volumes of D/S, DS NPs and SC@DS NPs treatment groups, the anti-tumor efficiency of SC-coated NPs on mice was better than that in free drugs and NPs group. Once the drug was prepared to the NPs and then functionalized with SC, the tumor inhibition efficacy was improved. These results showed that SC with good biosafety could be used as the vehicles for drug delivery and had an excellent protective effect on NPs, which can improve the stability of NPs in the complex GIT conditions and improve the anti-tumor effect.

Then the pathological features of small intestine and colon tissues were evaluated by the histology score changes according to the specific parameters (Additional file 1: Table S4). As shown in Fig. 5d, the hematoxylin and eosin (H & E) assay suggested that D/S and DS NPs could induce the inflammatory reaction such as focal lymphocytic infiltration; desquamation of epithelial cells and visible connective tissue hyperplasia in the lamina propri. However, there was no obvious inflammation was observed in the SC@DS NPs treatment group. Moreover, the groups based on the SC had almost negligible toxicity to the major organs (Additional file 1: Fig. S24), which indicated that the SC@DS NPs had a superior biosafety. Notably, the results of AB-PAS staining (Additional file 1: Fig. S25) and MPO immunofluorescence straining (Additional file 1: Fig. S26) further demonstrated that SC and SC-based preparation could not cause intestinal cellular infiltration and inflammation, which was consistent with the results of H&E straining. As depicted in Fig. 5e, the closely arranged tumor tissues and complete morphology of tumor cells were clearly observed in the saline and SC treatment groups. On the contrary, the shrinkage of nuclei and the reduction of the tumor cells density were observed in the D/S and DS NPs groups. Interestingly, the most notable necrosis of tumor cells was detected in SC@DS NPs treatment, which proved the fact that SC could prevent the degradation of DS NPs from the GIT conditions and improve the in vivo anti- tumor efficacy. To further investigate the therapeutic effect of different treatment, we performed TUNEL assay to evaluate the tumor apoptosis characteristics. As expected, large amounts of substantial apoptosis/necrosis were significantly observed in SC@DS NPs group, which suggested that SC@DS NPs could inhibit the tumor development via inducing apoptosis pathway (Fig. 5f). These anti-tumor results were also proved by the expression of apoptotic or anti apoptotic proteins in the tumor tissues *via* immunofluorescence analysis (Fig. 5g and Additional file 1: Fig. S27-30). These above results suggested that SC@DS NPs have a superior anti-tumor effect.

## Conclusion

In summary, learning from the biological characteristics of probiotic spores observed in nature, we rationally designed and developed a distinctive biomimetic spore nanoplatform (SC@DS NPs) to overcome both the mucosal diffusion barrier and the epithelial absorption barrier in one-stop. In this system, the SC integrated with multiple functions was served as a drug delivery vehicle. Compared with DS NPs, the SC@DS NPs retained the characteristic of probiotic spores, which could resist the extreme stomach acid environment after oral administration and subsequently deliver to the intestine successfully. Our study demonstrated that the NPs coated with SC were of lower mucin affinity and superior mucus penetration capability. This result was attributed to that the SC is rich in sulfhydryl groups that could cleave the disulfide bonds between mucus glycoproteins. We found that the intestinal epithelial absorption efficiency of SC@DS NPs was dramatically improved due to the SC containing some specific proteins. We demonstrated that such biomimetic spore nanoplatform can successively overcome the mucosal diffusion barrier and the epithelial absorption barrier only by coating SC on the surface of NPs.

### Electronic supplementary material

Below is the link to the electronic supplementary material.

Supplementary Material 1
