# Supplementary material for: A multifunctional integrated biomimetic spore nanoplatform for successively overcoming oral biological barriers
Source: J Nanobiotechnology. 2023 Aug 29;21:302. doi: 10.1186/s12951-023-01995-z (PMC10463901; doi:10.1186/s12951-023-01995-z)
Supplement: Supplementary file 1 — Supplementary Material 1 [file 12951_2023_1995_MOESM1_ESM.docx]

Supporting Information

Title

**A multifunctional integrated biomimetic spore nanoplatform for successively overcoming oral biological barriers**

Qingling Song^a,b,c,1^, Junfei Yang^a,b,1^, Xiaocui Wu^a,b^, Jiannan Jiao^a,b^, Hongjuan Zhao^a,b,c^, Qianhua Feng^a,b,c^, Zhenzhong Zhang^a,b,c,^*, Yun Zhang^a,b,c,^* and Lei Wang^a,b,c,^*

^a^School of Pharmaceutical Sciences, Zhengzhou University, Zhengzhou 450001, People’s Republic of China

^b^Henan Key Laboratory of Targeting Therapy and Diagnosis for Critical Diseases, Zhengzhou 450001, People’s Republic of China

^c^Key Laboratory of Advanced Drug Preparation Technologies, Ministry of Education, Zhengzhou 450001, People’s Republic of China

E-mail: zhangzhenzhong@zzu.edu.cn (Z. Z. Zhang), zhang_yun@ymail.com (Y. Zhang) and wanglei1@zzu.edu.cn (L. Wang)

^1^These two authors contributed equally to this work.

**Experimental Section**

***Materials*:** Doxorubicin hydrochloride (DOX) and porcine gastric mucin were purchased from Dalian Meilun Biotechnology Co. LTD. Sorafenib (SOR) and pravastatin sodium (Pra) were obtained from Aladdin Chemistry Co., Ltd (Shanghai, China) and Macklin Biochemical Co., Ltd (Shanghai, China), respectively. Methyl-β-cyclodextrin (M-β-CD) was purchased from MedChemExpress (Monmouth Junction, NJ, USA). Chlorpromazine, amiloride, indometacin, lovastatin and wheat germ agglutinin labeled with FITC fluorescein were all obtained from Sigma Aldrich, Inc. (St Louis, MO, USA). *Bacillus coagulans* (BC) was purchased from BeNa Chuang Lian Biotechnology Research Institute (Beijing, China).

***Preparation of spore capsid (SC):*** Firstly, we cultured BC in the sporulation medium and then separated the pure spores referring to the previously reported method [1]. Next, the spores were dispersed in the sodium chloride solution with high concentration (20% NaCl), and incubated for another time to release their contents, which was attributed to the osmotic pressure difference inside and outside of spores. Finally, the complete spore capsid (SC) was obtained by centrifugation at 3000 × *g* for 20 min and followed by washing with deionized water.

***Synthesis of DS NPs:*** DS nanoparticles (DS NPs) were fabricated according to the previously reported method with slight modification [2]. Firstly, sorafenib (SOR) was dissolved in DMSO (10 mg·mL^-1^) and then added dropwise (20 µL/15 s) to the mixture solution containing doxorubicin hydrochloride (DOX, 1 mg mL^-1^) and 0.05 mM sodium bicarbonate at the ratio of 1: 6 (*v/v*) under slight shaking. Subsequently, the mixture solution was centrifuged (12000 × *g*, 20 min) and washed with deionized water for 3 times. Finally, the precipitate was re-suspended and lyophilized for further use. The drug loading efficiency (DL) of DOX and SOR in DS NPs was calculated according to the following formula:

$$DL (\%)=\frac{M_{drug-loaded}}{M_{drug-loaded}+M_{nanoparticles}}\times100\%$$

***Synthesis of SC@DS NPs:*** For wrapping SC to the surface of DS NPs, the SC was firstly broken by Ultrasonic Homogenizer (JY92-IIN, Scientz). And then the SC debris and DS NPs mixture solution were added to the liposome extruder (LiposoEasy LE-1, MORGEC, America) at an appropriate volume ratio and extruded back and forth for 13 times through the polycarbonate membrane (pore size: 800 nm, 400 nm). Then the mixture solution was centrifuged at 10000 × *g* for 10 min and followed by washing with deionized water to obtain SC@DS NPs.

***Characterization:*** The morphologies of spores, SC, DS NPs and SC@DS NPs were carried out on a 150kV Transmission Electron Microscope (TEM, Hitachi, HT7700, Japan) and  a Scanning Electron Microscope (SEM, Hitachi, SU8010, Japan), respectively. And the appearance of different preparations was observed by photos. The size of DS NPs and SC@DS NPs was evaluated by Nanoparticle Tracking Analysis (NTA, Nanosight NS300, UK) and Dynamic Light Scattering (DLS, Zetasizer Nano ZS-90, Malvern, UK). The zeta potential was conducted by using a Zetasizer Nano ZS (Malvern). The proteins of SC were analyzed by LCMSMS (nanoLC-QE). In order to demonstrate whether SC coating process influences the surface composition of SC, the total proteins of SC@DS NPs and SC were extracted and further analyzed by Gel imaging system (Gel DocTM XR+, BIO RAD, America), respectively.

***Evaluation of stability:*** In order to investigate the stability of DS NPs and SC@DS NPs *in vitro*, they were incubated in the simulated gastric fluid (SGF) for 2 h, respectively, and their morphologies were carried out by TEM. Then their stability was also evaluated by the changes in size with time prolongation. Moreover, the drug release of DS NPs and SC@DS NPs was also determined after being incubated in SGF and stimulated intestinal fluids (SIF) for 4 h at 37 °C, respectively. Subsequently, the release characteristic of DS NPs and SC@DS NPs was further evaluated in stimulated blood (pH 7.4) and stimulated tumor environment (pH 5.5) for further 20 h, respectively. The amount of released DOX and SOR was subsequently analyzed by the Fluorescence Spectrophotometer (RF-5301PC, Japan) and by using High Performance Liquid Chromatography (HPLC, Waters 2960, USA), respectively. Additionally, the stability of SC@DS NPs in PBS, serum and cell culture media also was evaluated.

***Mucin aggregation:*** To further evaluate whether the NPs could escape from the mucus trap, we incubated free D/S (free DOX + SOR), DS NPs and SC@DS NPs in the 0.5% porcine mucin solution, respectively. The samples were subsequently mixed enough by vortex for 1 min, and continued to incubate in the shaker for 30 min (100 rpm, 37 °C). After incubation, the mixture samples were centrifuged (1000 × *g*, 5 min) to obtain the mucin-NPs aggregates. Then the aggregates were disintegrated with the solution containing DMSO and PBS at the ratio of 3: 1 (*v/v*), and the fluorescence intensity of DOX was measured by Microplate reader (Synergy H1, Bio Tek, USA). To explore the mucus penetration ability of NPs, we also performed *in vitro* cell internalization study using a mucus-secreting cell line [3].

***Cell culture:*** The Caco-2 cell line was purchased from Procell Life Science&Technology Co.,Ltd. We cultured Caco-2 cells in MEM medium (Procell, PM150410, China) supplemented with 20% fetal bovine serum (FBS). The human colon cancer cell line (SW620) was grown in DMEM/high glucose (BI Biological Industries) added with 10% FBS and 100 μg·mL^-1^ streptomycin and 100 U·mL^-1^ penicillin (Solarbio). These two cell lines were seeded in the culture flask and cultured in a humidified incubator at 37 °C and 5% CO_2_, respectively.

***Cellular Uptake Mechanism:*** For the study of cellular uptake, Caco-2 cells were seeded in 6-well plate with 3 × 10^5^ cells each well. After culture for 48 h, the Caco-2 cells were incubated in the fresh medium containing DS NPs and SC@DS NPs at the concentration of 5 μg mL^-1^ DOX for 2 h and 4 h, respectively. The cell uptake rate was determined by Confocal Laser Scanning Microscopy (CLSM, Leica TCS SP8*, Germany) and Flow Cytometry (BD Accuri C6, America). In order to explore the role of oligopeptide transporter and monocarboxylate transporter-1 (MCT1) in endocytosis, the related inhibitors such as glycyl-sarcosine (Gly-sar) and pravastatin (Pra) were employed to incubate with cells for 30 min, which was prior to the subsequent incubation with different preparations. After the preset incubation time, NPs and inhibitors were removed and followed by washing the cells with PBS. Then the amount of uptake was performed as the described method in the above cellular uptake experiment. To further explain the related cellular uptake mechanism, we selected several inhibitors according to previous reports [4, 5]. Specifically, the endocytosis inhibitors such as chlorpromazine, amiloride, indometacin, lovastatin and methyl-β-cyclodextrin were pre-incubated with Caco-2 cells for 30 min, respectively, and followed by treatment with DS NPs and SC@DS NPs for 4 h at the presence of inhibitors. The relative amount of uptake was detected as described above.

***Transepithelial transport study:*** For investigating the transcellular transport of DS NPs and SC@DS NPs, Caco-2 cells were seeded in transwell chamber with polycarbonate membranes (Corning Incorporated, Costar). After culture for 21 days, the transepithelial electrical resistance (TEER) values were measured to explore whether the cell monolayer was established successfully. Subsequently, the apical solution was replaced by the fresh growth medium containing free DOX, DS NPs and SC@DS NPs at the concentration of 5 μg mL^-1^ DOX for 2 h. Then, the transepithelial transport of different groups could be visualized with CLSM. Additionally, TEER values were also performed to confirm the cell monolayer integrity. The expression level of Occludin after the Caco-2 cells being incubated with different groups was determined by immunofluorescence staining. And the transepithelial transport amount of DS NPs and SC@DS NPs was measured in the basolateral chambers by the method as described above. Simultaneously, the morphologies of the DS NPs and SC@DS NPs were evaluated by TEM images.

***In vitro cytotoxicity***: For the study of *in vitro* cytotoxicity, the Caco-2 cell line and colon cancer cell line (SW620) were seeded in the 6-well plate, respectively. Firstly, the cytotoxicity of SC and SC@DS NPs in Caco-2 cells was evaluated. Then, the SW620 cells were treated with free DOX, DS NPs and SC@DS NPs for 2 h and 4 h, respectively. The cell uptake rate on SW620 was analyzed by CLSM and Flow Cytometry. Moreover, to analyze the cell apoptosis, we also treated the cells with different groups for 24 h and detected by Annexin V-FITC/PI apoptosis kit. Moreover, after incubation, the cells were also harvested and analyzed by Flow Cytometry.

***Western Blot:*** SW620 cells were treated with D/S, DS NPs and SC@DS NPs as prescribed for 24 h. Total proteins were extracted for Western blot by RIPA lysis buffer. Anti-Bax (Abcam, ab32503), anti-Bcl-2 (Abcam, ab182858), Anti-cleaved Caspase-3 (CST, Asp175) and Anti-Caspase-9 (Abcam, ab202068) were served as primary antibodies. Finally, multifunctional gel imaging (BioSpectrum®615 Imaging System, UVP, USA) was used to detect the expression of these target proteins.

***Animals:*** BALB/c and BALB/c nude mice (female, 6-8 weeks) were purchased from SPF (Beijing) Biotechnology Co., Ltd. All the work performed on animals was in accordance with the Guidelines for Care and Use of Laboratory Animals of Zhengzhou University, and the experiments were approved by the Animal Ethics Committee of Zhengzhou University.

***Mucus penetration in vivo:*** BALB/c mice were fasted for 12 h with free access to water prior to the experiment. Firstly, we anesthetized the mice and ligated their jejunum, then injected 0.2 mL of free DOX, DS NPs and SC@DS NPs (containing DOX: 15μg/mL and wheat germ agglutinin: 10 μg/mL) into the intestinal cavity, respectively. Next, we ligated the other end of the small intestine and put it back into the abdominal cavity. After 1 h incubation, the intestinal tissues were harvested and fixed on the slide and cryostat sectioning was performed, respectively. Finally, the 2D mucus coverage and 3D mucus-penetrating effect of different groups were determined by CLSM. We next investigated whether the differences are attributed to alterations in trap or permeability of NPs at the mucosal surface. The different groups were directly administered into the small intestine of mouse, and then the intestinal tissues were excised, opened and flattened for visualization 30 min after administration. The images were obtained by CLSM.

***Intestinal absorption and circulat******ion in situ:*** In order to evaluate the intestinal absorption of different formulations, mice were orally administered with D/S, DS NPs, SC@DS NPs, SC@DS NPs + Pra and SC@DS NPs + Gly-sar at a dose of 30 mg/kg of DOX, respectively. Among these groups, the Pra and Gly-sar were pre-treated for 30 min. After 4 h administration, the intestinal tissues were collected and stained with DAPI. The red fluorescence of DOX was detected by CLSM. Moreover, the circulation of different NPs was investigated by the *in situ* intestinal circulation method according to the previously report [1]. The drug absorption in the intestine was estimated by measuring drug concentration in the circulating solution at preset time points.

***In vivo pharmacokinetic study:*** Female Sprague-Dawley rats (200-220 g) were fasted for 12 h with free access to water. Prior to the experiments, the rats were randomly divided into 5 groups (*n* = 3). The rats were treated with free D/S, DS NPs, SC@DS NPs, SC@DS NPs + Pra and SC@DS NPs + Gly-sar at a dose equivalent to 30 mg/kg of DOX, respectively. Then, the blood samples were obtained at preset time points. The plasma samples were separated by centrifugation (1000× *g*, 5 min), and analyzed by using HPLC (Waters 2960, USA) according to the previously reported method [6]. The mobile phase was consisted of methanol, acetonitrile and 1% ethylic acid (38 :32 :30, V/V/V) at the flow rate of 1 mL·min^-1^.

***Establishment of colon cancer models:*** The human colon cell line SW620 of 1 × 10^7^ cells were inoculated subcutaneously to the BALB/c nude mice. During the model establishment, the tumor volume of each mouse was monitored and calculated by the formula: (L × W^2^)/2, where L and W refer to the length and width of the tumor, respectively.

***In vivo distribution:*** For visualizing the *in vivo* distribution of different NPs, IR783 was used to replace DOX to prepare the self-assembled IR783 NPs. Then the Free IR783, IR783 NPs and SC@IR783 NPs were administrated to the tumor-bearing mice at the equivalent IR783 dose of 2 mg/kg, respectively. At preset times, the fluorescence images were captured using the *In Vivo* Imaging System (Bruker, Germany). Additionally, the *in vivo* distribution in important organs at 2 h, 4 h, 6 h, 8 h, 10 h and 12 h was also evaluated.

***In vivo pharmacodynamics evaluation:*** The tumor-bearing mice were randomly assigned into 5 groups (*n* = 6). Then they were orally treated with saline, D/S, SC, DS NPs and SC@DS NPs every day, respectively. The tumor volume and body weight of each mouse were recorded every 2 days. At the end of treatment, the major tissues were obtained for haematoxylin and eosin (H & E) staining. Among these tissues, histology score was used to evaluate the pathological damage degree of intestine and colon regions. And the H & E staining and terminal deoxynucleotidyl transferase-mediated dUTP nick-end labeling (TUNEL) were performed to analyze the level of tumor tissue apoptosis. AB-PAS staining and MPO staining were used to evaluate the pathological features of colonic tissues among all treatment groups. Furthermore, immunofluorescence was also used to analyze the tumor apoptosis related proteins such as Caspase-3, Caspase-9, Bax and Bcl-2. The expression level of these proteins was further semi-quantitatively analyzed by Image J software.

References

1. Song Q, Zheng C, Jia J, Zhao H, Feng Q, Zhang H, Wang L, Zhang Z, Zhang Y: **A Probiotic Spore-Based Oral Autonomous Nanoparticles Generator for Cancer Therapy.** *Advanced Materials* 2019, **31:**e1903793.

2. Shamay Y, Shah J, Isik M, Mizrachi A, Leibold J, Tschaharganeh DF, Roxbury D, Budhathoki-Uprety J, Nawaly K, Sugarman JL, et al: **Quantitative self-assembly prediction yields targeted nanomedicines.** *Nature Materials* 2018, **17:**361-368.

3. Zhu X, Wu J, Shan W, Zhou Z, Liu M, Huang Y: **Sub-50 nm Nanoparticles with Biomimetic Surfaces to Sequentially Overcome the Mucosal Diffusion Barrier and the Epithelial Absorption Barrier.** *Advanced Functional Materials* 2016, **26:**2728-2738.

4. Wu L, Liu M, Shan W, Zhu X, Li L, Zhang Z, Huang Y: **Bioinspired butyrate-functionalized nanovehicles for targeted oral delivery of biomacromolecular drugs.** *Journal of Controlled Release* 2017, **262:**273-283.

5. Cui Y, Shan W, Liu M, Wu L, Huang Y: **A strategy for developing effective orally-delivered nanoparticles through modulation of the surface “hydrophilicity/hydrophobicity balance”.** *Journal of Materials Chemistry B* 2017, **5:**1302-1314.

6. Wang L, Shi J, Jia X, Liu R, Wang H, Wang Z, Li L, Zhang J, Zhang C, Zhang Z: **NIR-/pH-Responsive drug delivery of functionalized single-walled carbon nanotubes for potential application in cancer chemo-photothermal therapy.** *Pharm Reserach* 2013, **30:**2757-2771.

**Supplementary Figures**


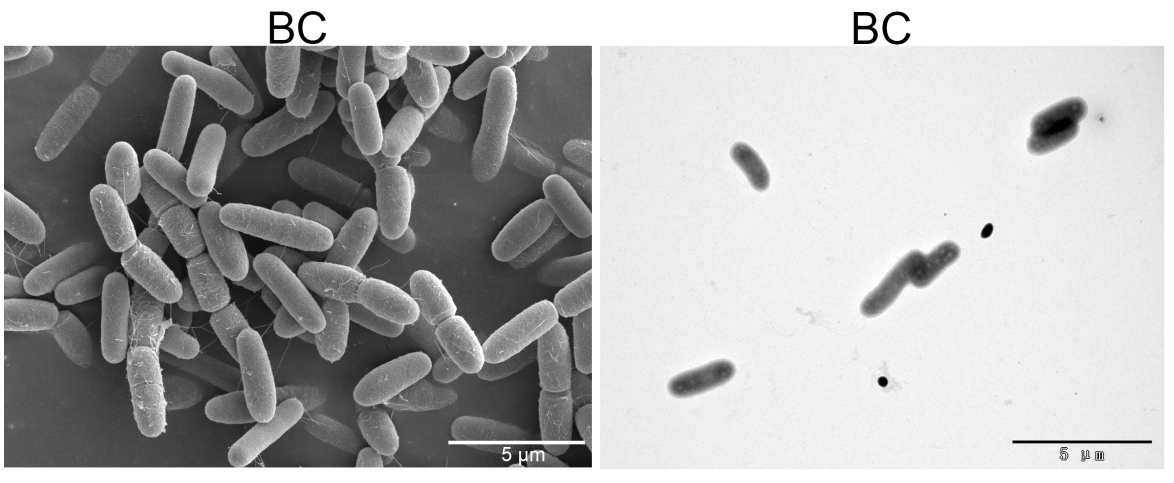


1. SEM and TEM images of BC.


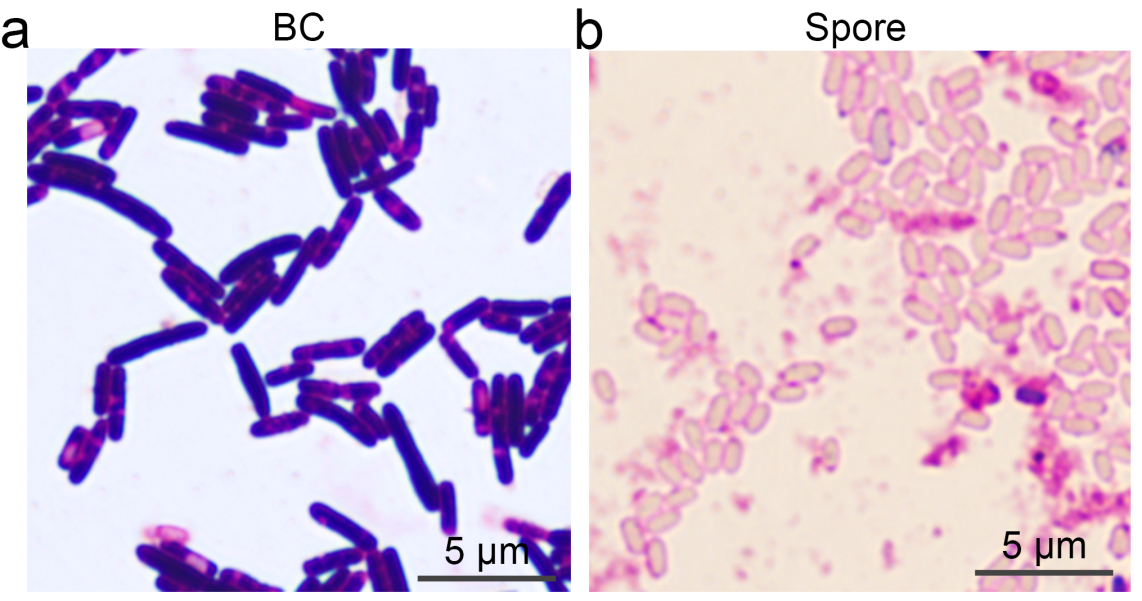


1. Gram straining images of a) BC and b) spore.


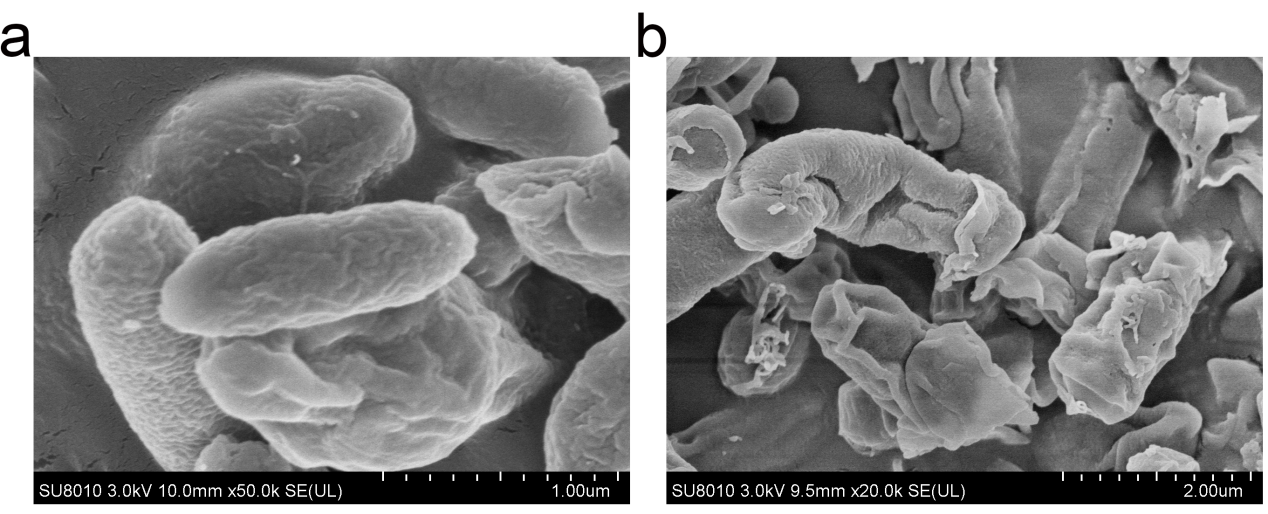


1. Spores were incubated in the sodium chloride solution (NaCl) with a) low concentration (5%) and medium concentration (10%) for 2 h.


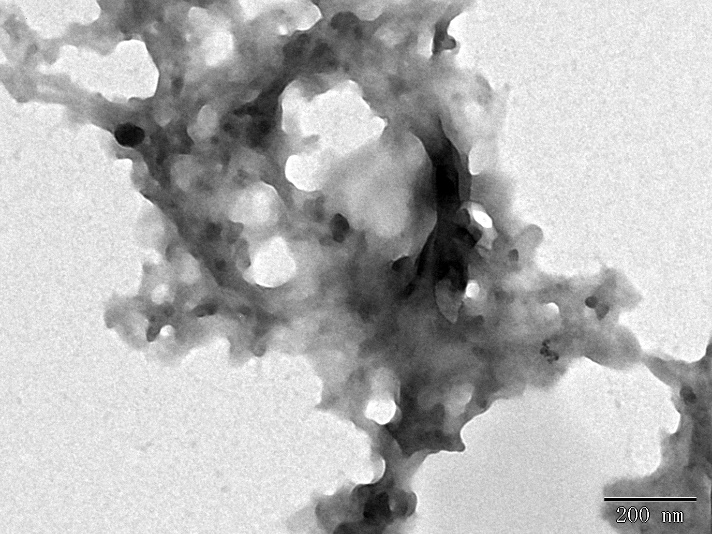


1. The TEM image of SC debris.


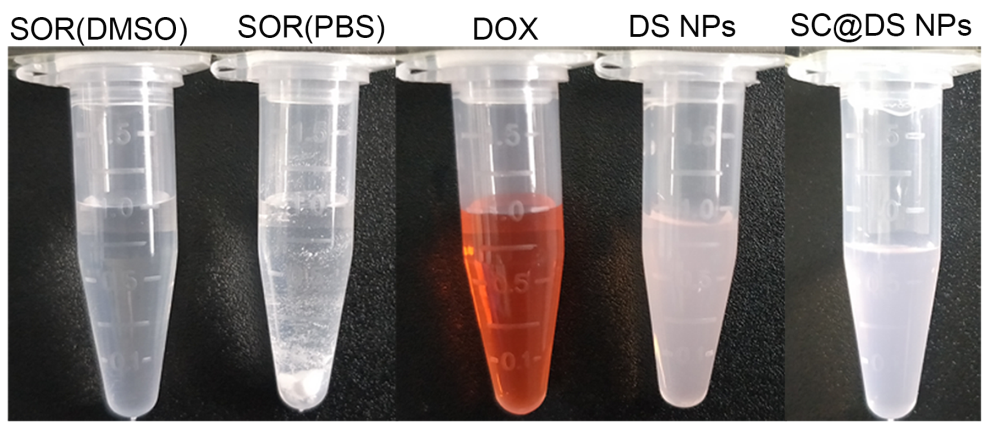


1. The photograph of different preparations.


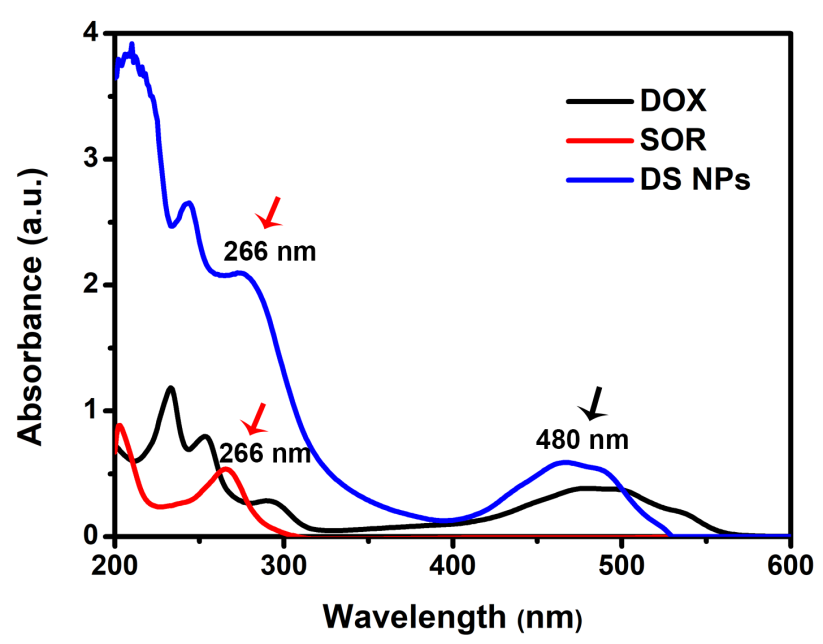


1. The UV-vis spectrogram of DOX, SOR and DS NPs.


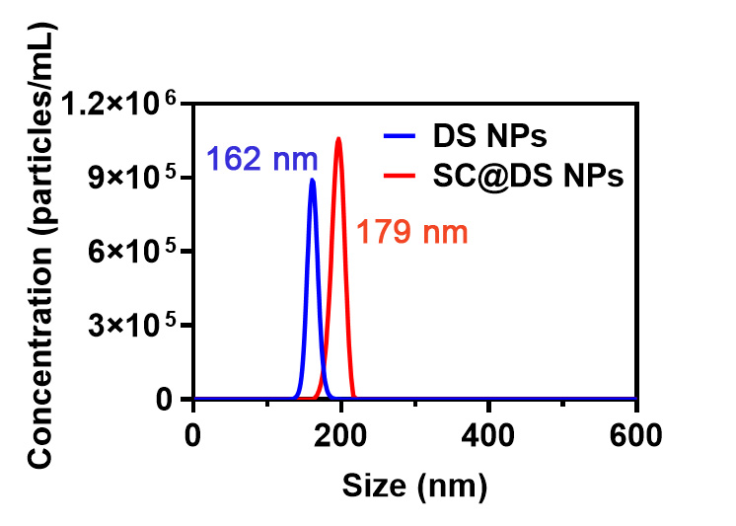


1. Evaluation of size of DS and SC@DS NPs by using NTA.


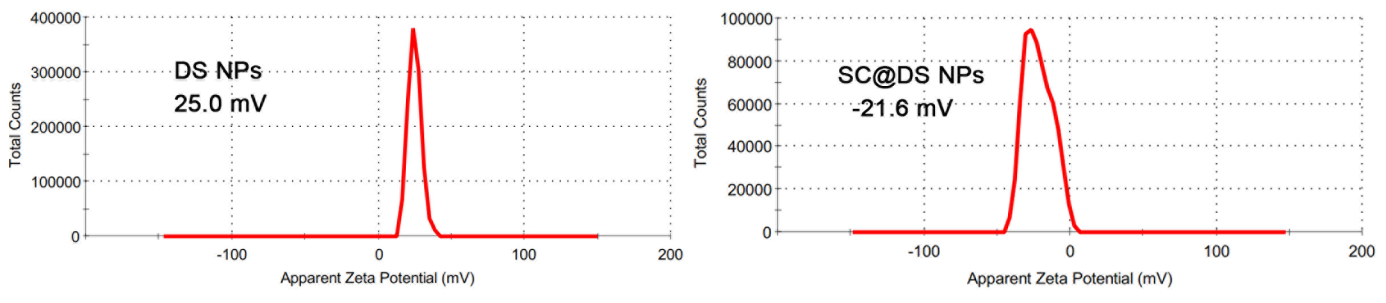


1. The zeta potential of DS NPs and SC@DS NPs.


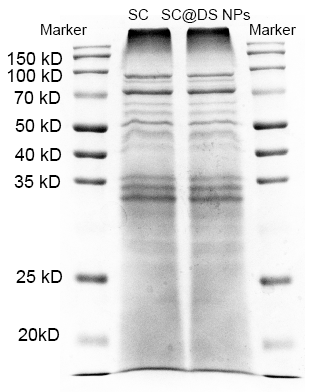


1. The protein components of SC and SC@DS NPs determined by Gel imaging.


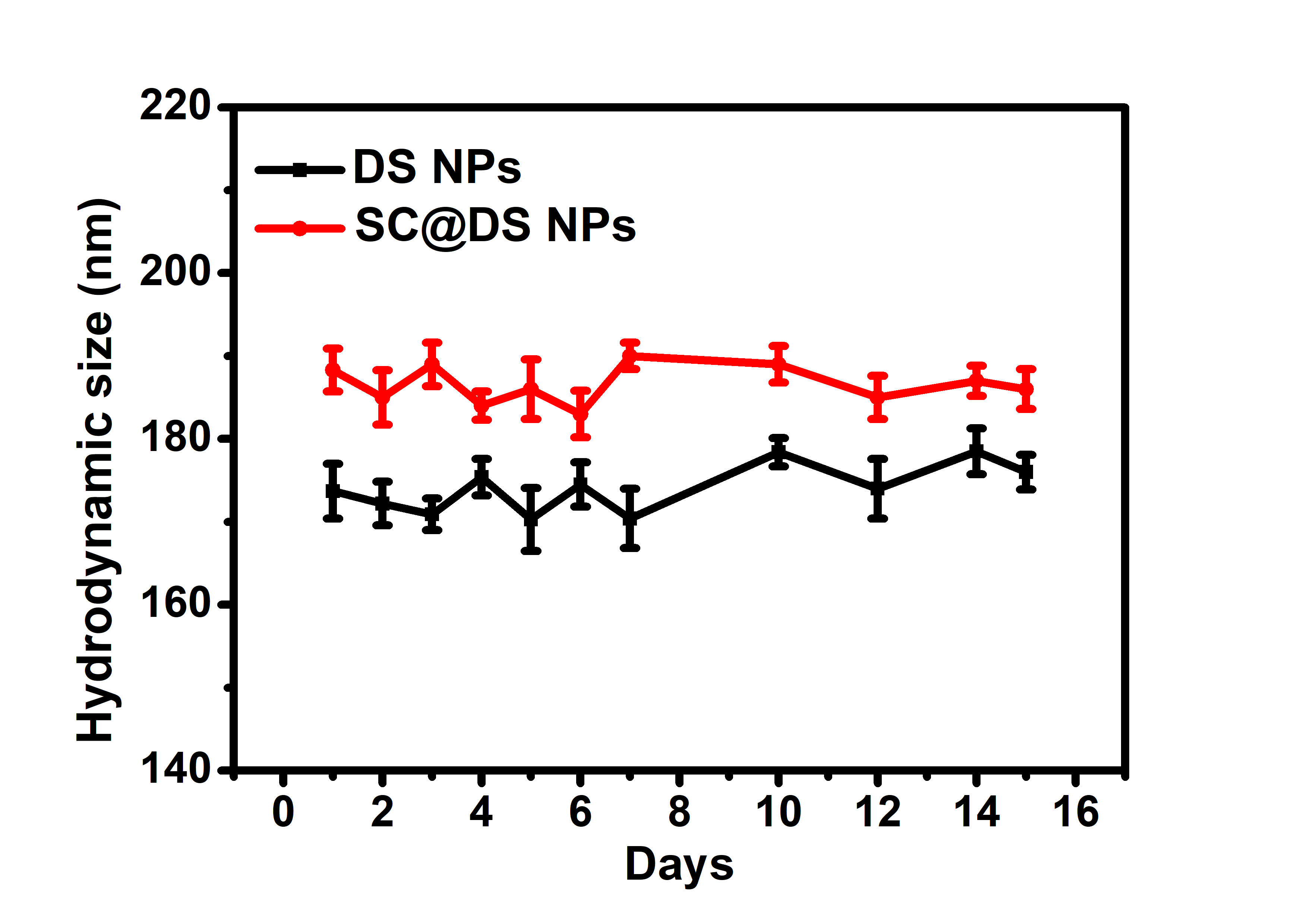


1. Stability of DS NPs and SC@DS NPs dispersed in PBS detected by DLS.





1. Stability evaluation of SC@DS NPs dispersed in PBS detected by NTA at the room temperature.


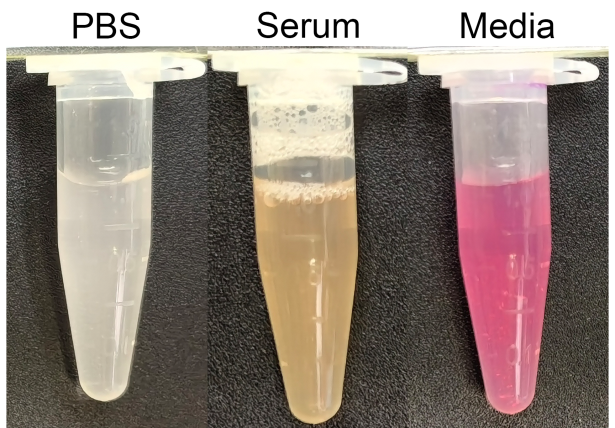


1. Stability of SC@DS NPs dispersed in PBS, serum and cell culture media.


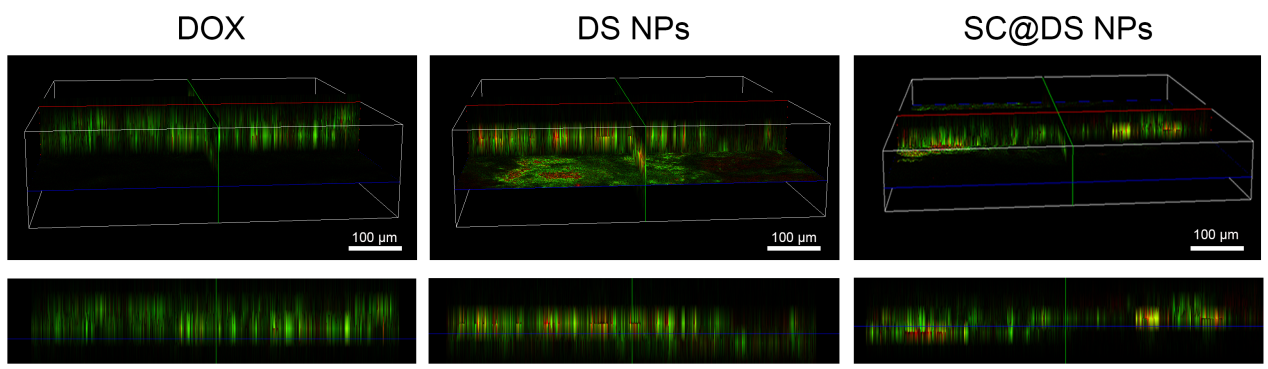


1. Vertical distributionof NPs (red) on E12 cell monolayer; mucus was stained with wheat germ agglutinin straining (green).


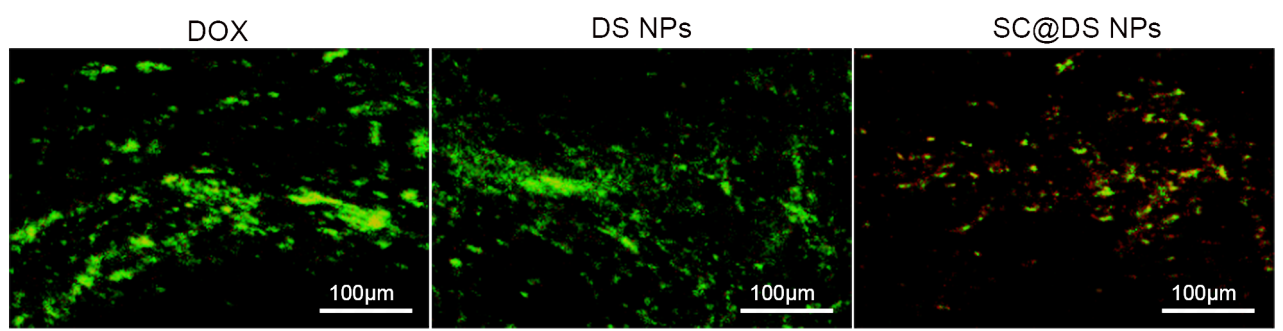


1. Representative images of NPs distribution (red) on the mucosal surface (green).


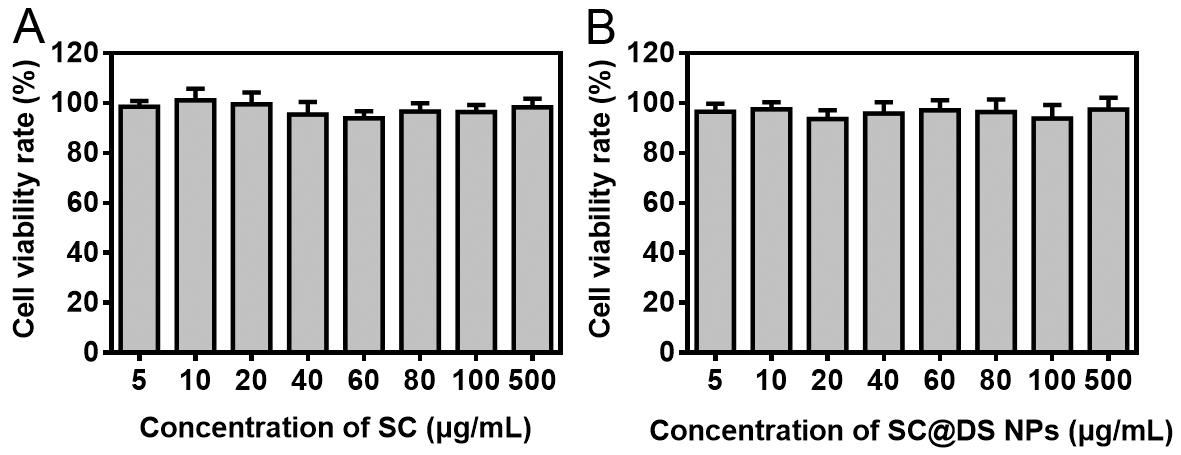


1. Cell viability rate of SC and SC@DS NPs in Caco-2 cells at different concentrations (n = 6).


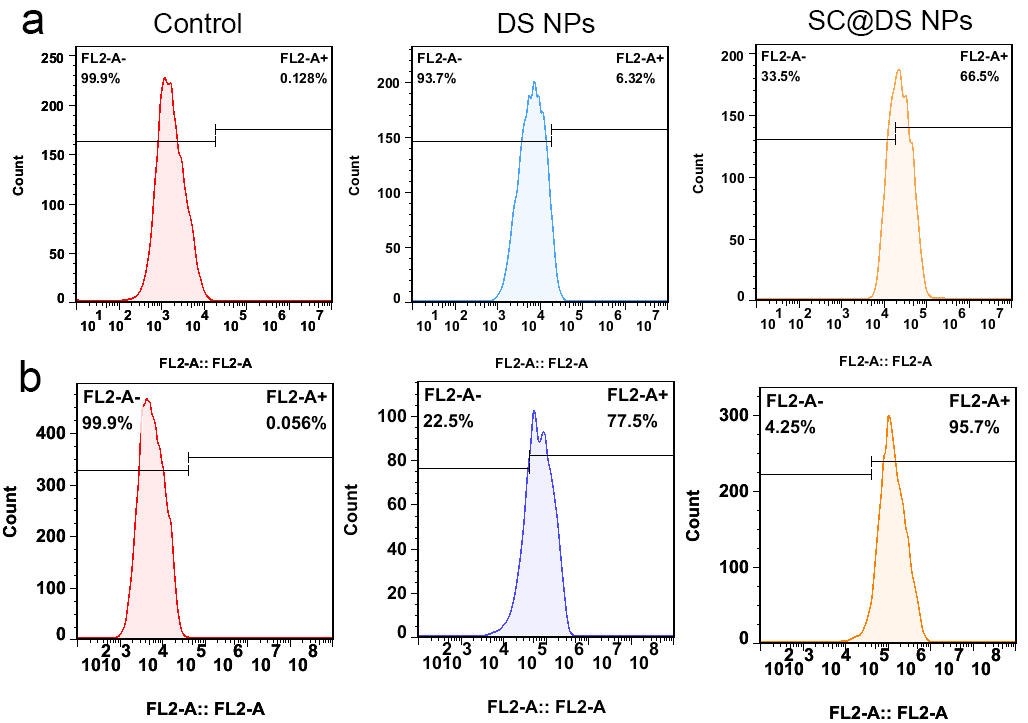


1. The relevant representative flow cytometry profiles after the Caco-2 cells being incubated with DS NPs and SC@DS NPs for a) 2 h and b) 4 h, respectively.


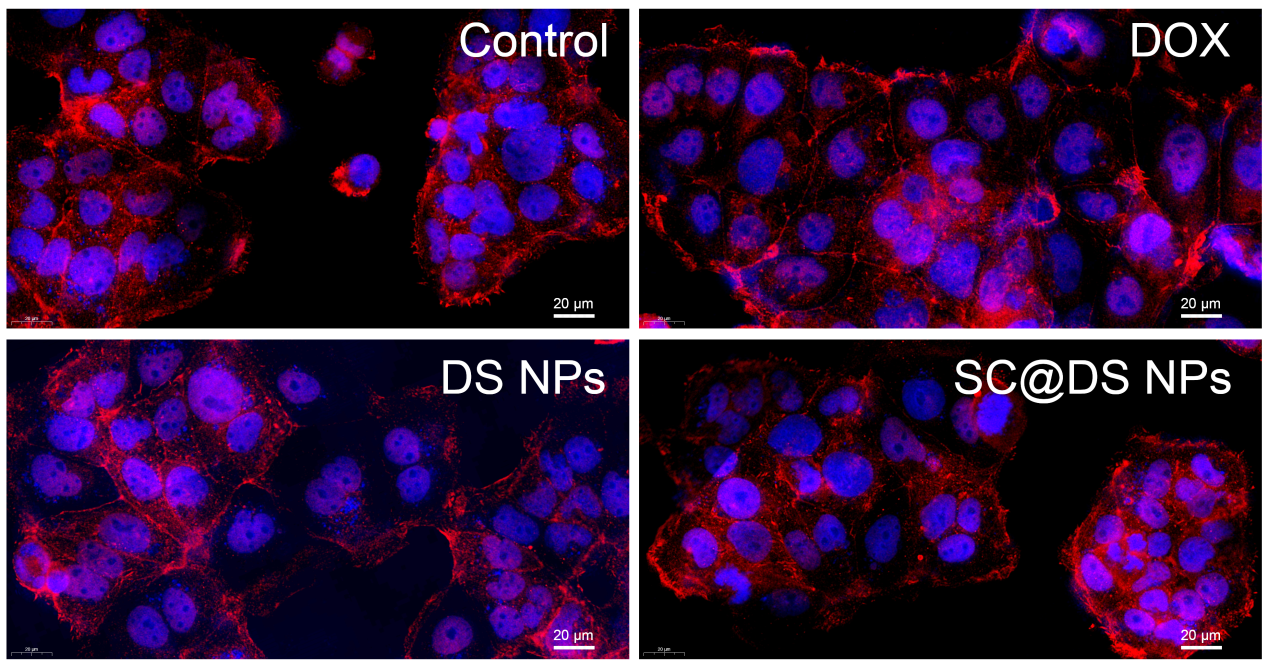


1. Immunofluorescence staining of Caco-2 cells was used to evaluate the expression level of tight junction protein Occludin (red fluorescence).


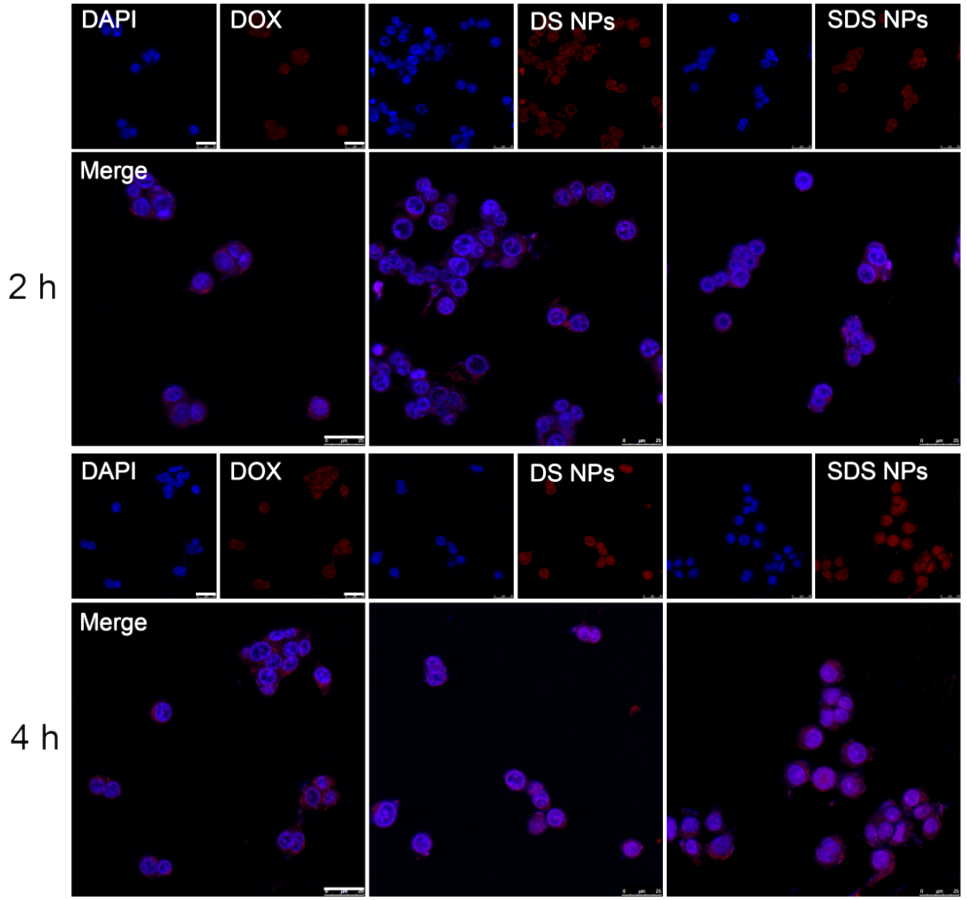


1. Cellular uptake of NPs in SW620 tumor cells. The Caco-2 cells were incubated with free DOX, DS NPs and SC@DS NPs for 2 h and 4 h, respectively. Scale bar: 25 μm.


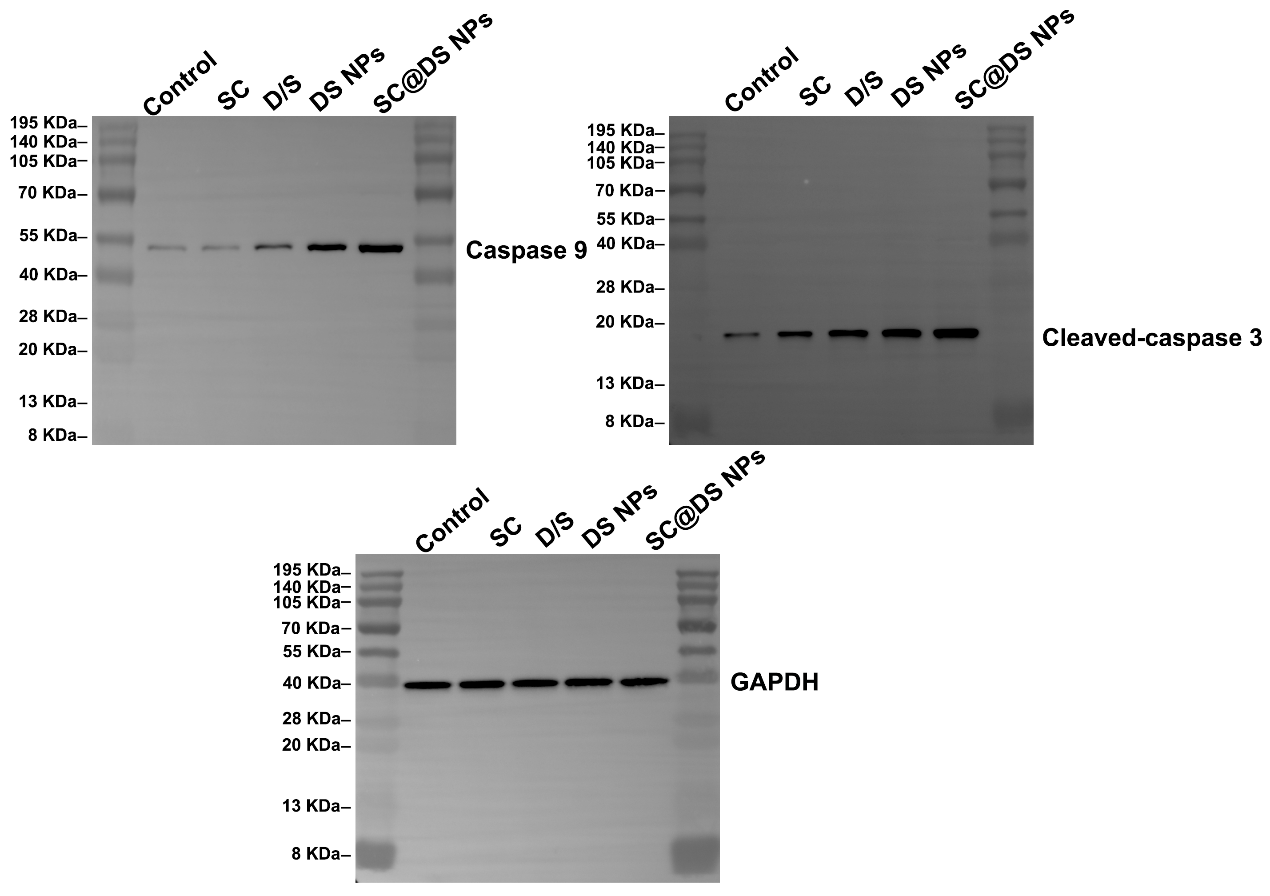


1. The uncropped blot images of Fig. 3c.


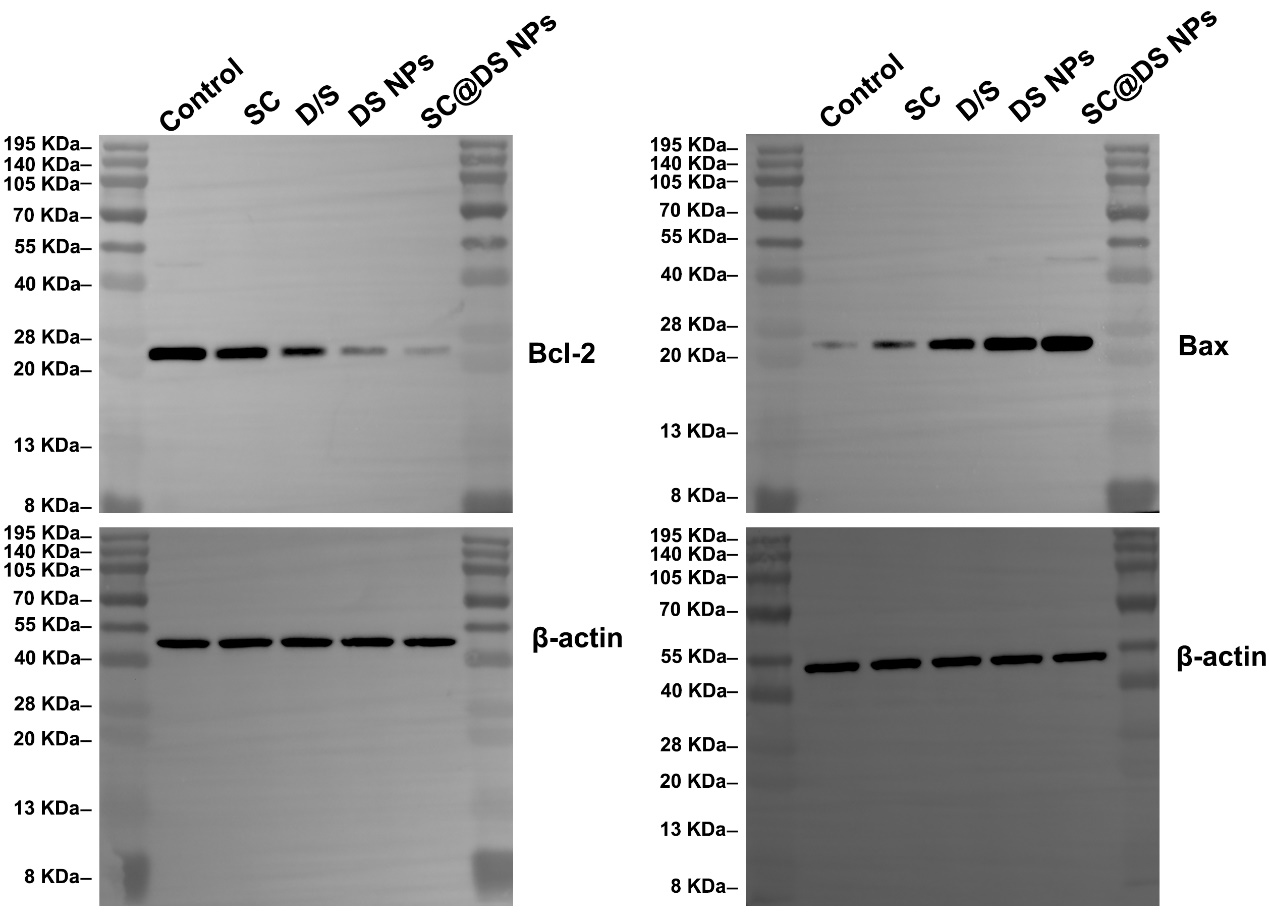


1. The uncropped blot images of Fig. 3e.


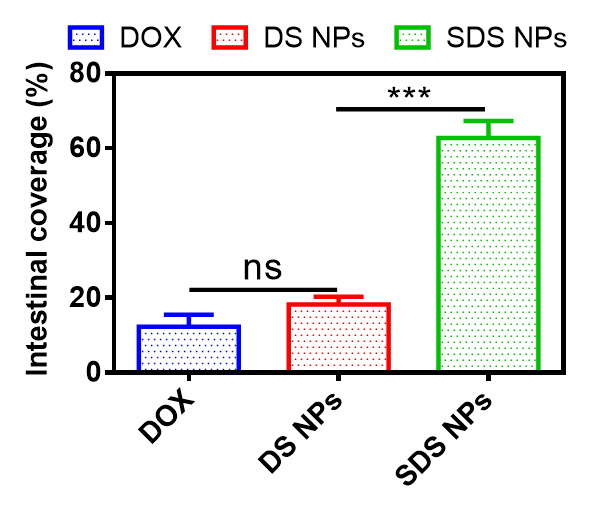


1. Quantification of the NPs coverage in the mucus.


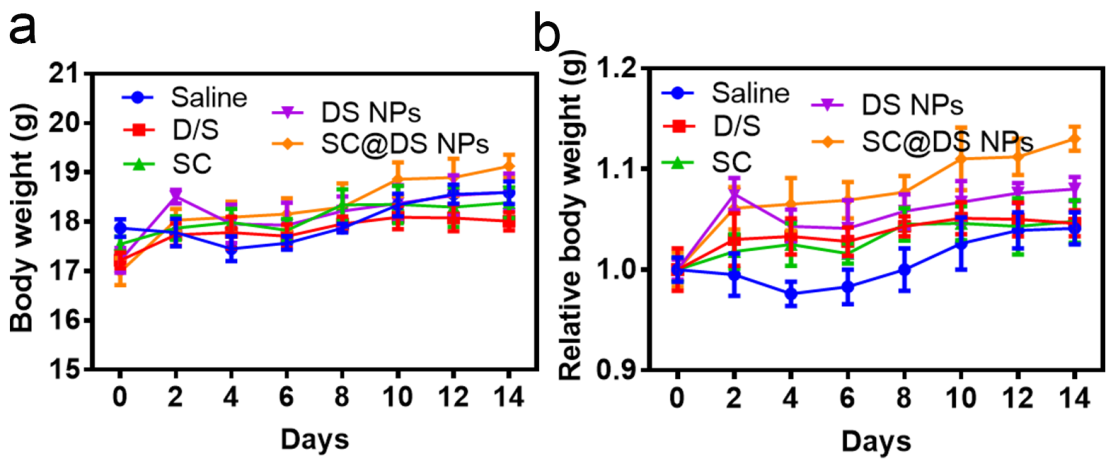


1. a) Changes in body weight during treatment and b) the relative body weight at the end of different treatment.


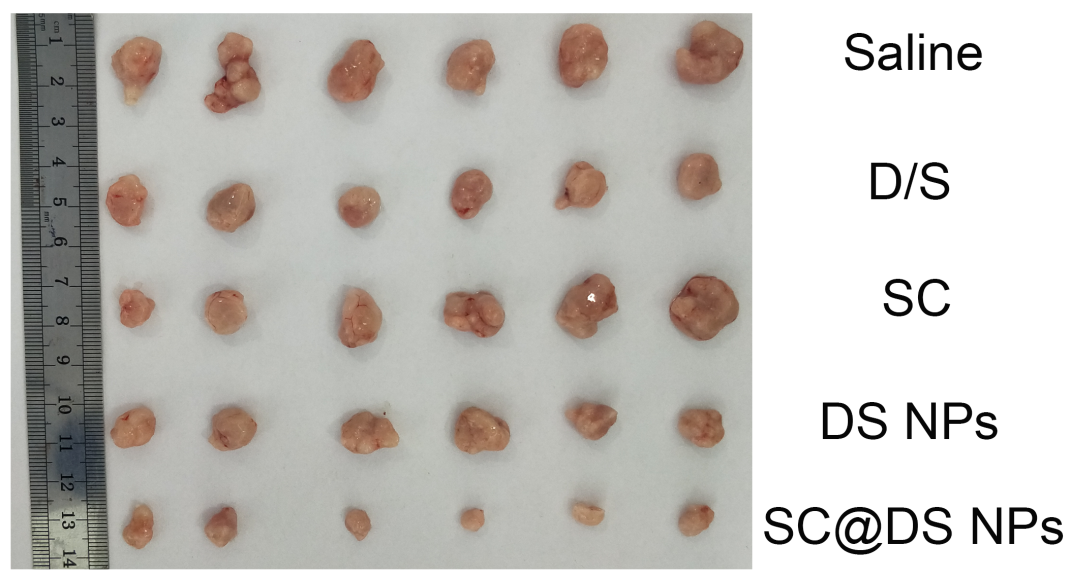


1. Representative pictures of tumor tissues of each mouse after different treatments.


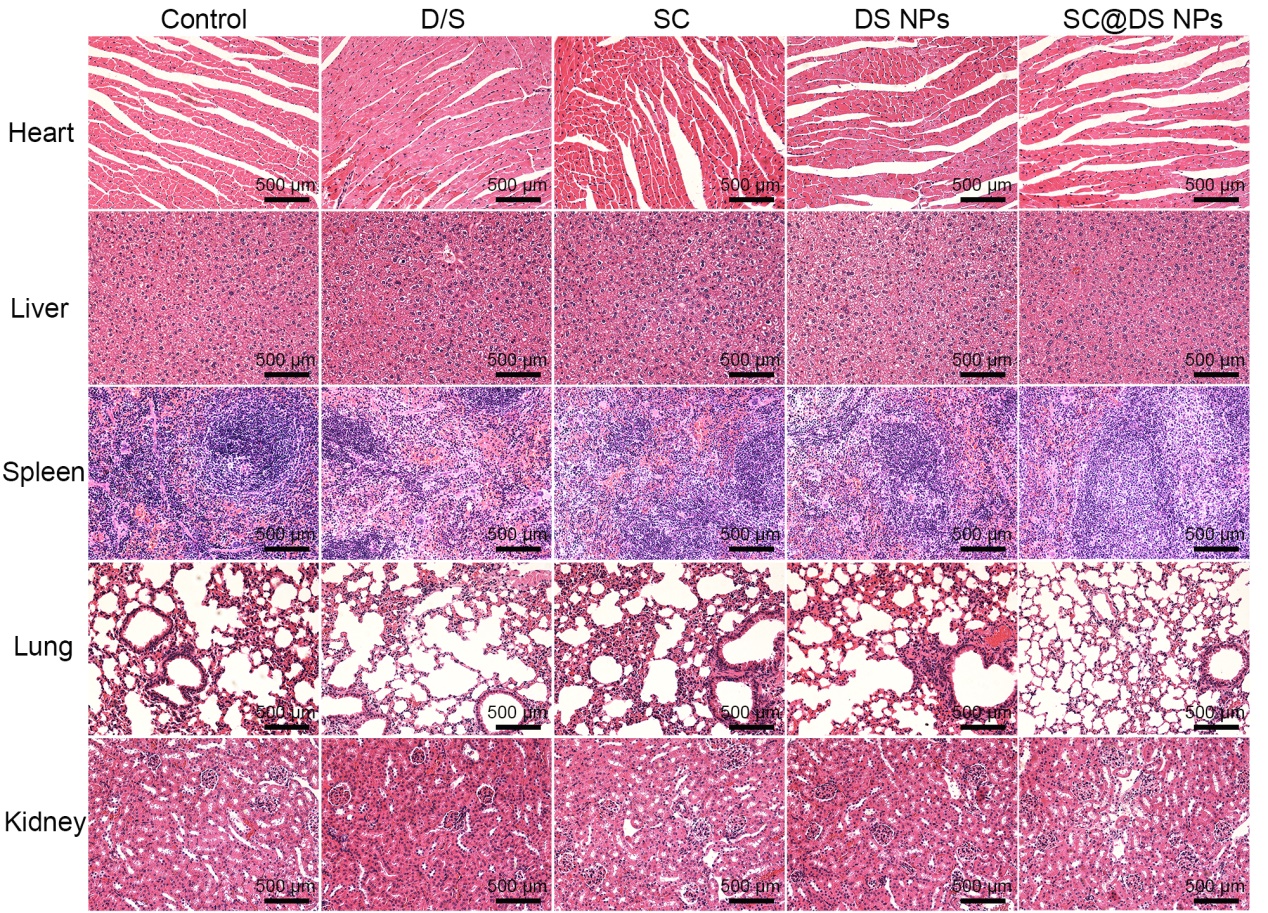


1. Histologic assessments of major organs with H&E staining after the mice being treated with different groups.


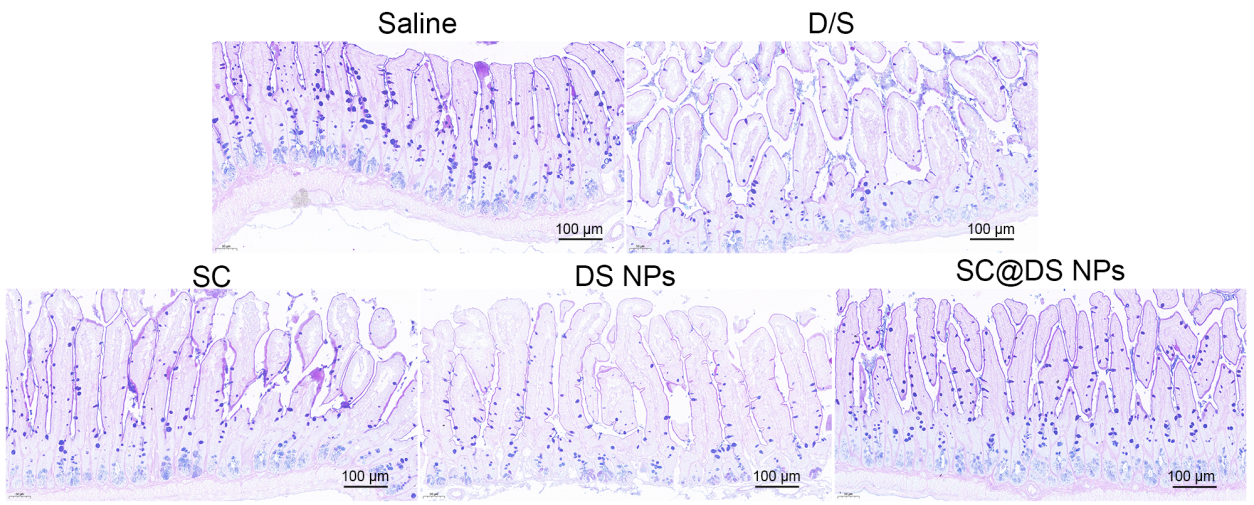


1. AB-PAS staining for colonic goblet cells after different treatment.


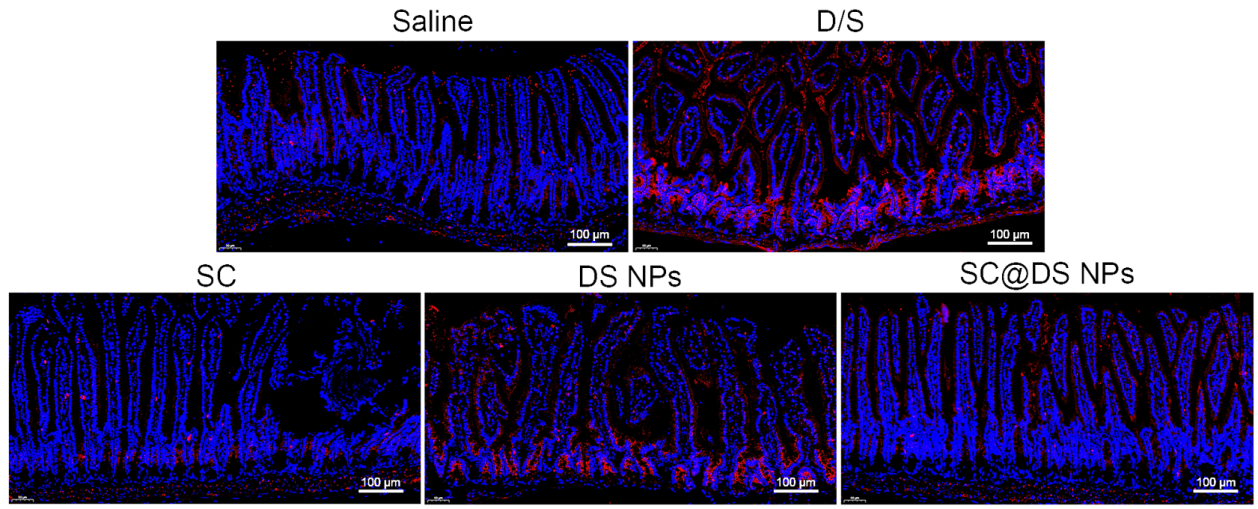


1. MPO staining for colonic tissues after different treatment.


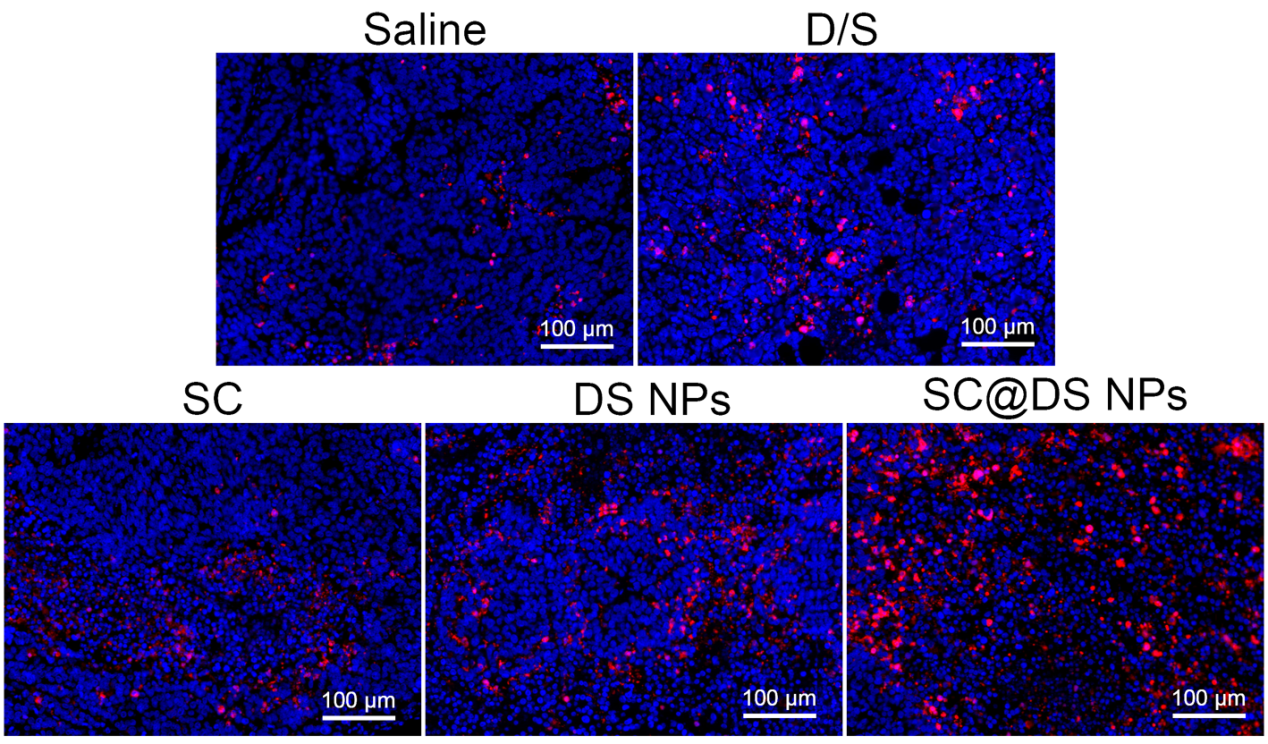


1. The protein expression of caspase 3 in tumor tissues by the immunofluorescence analysis.


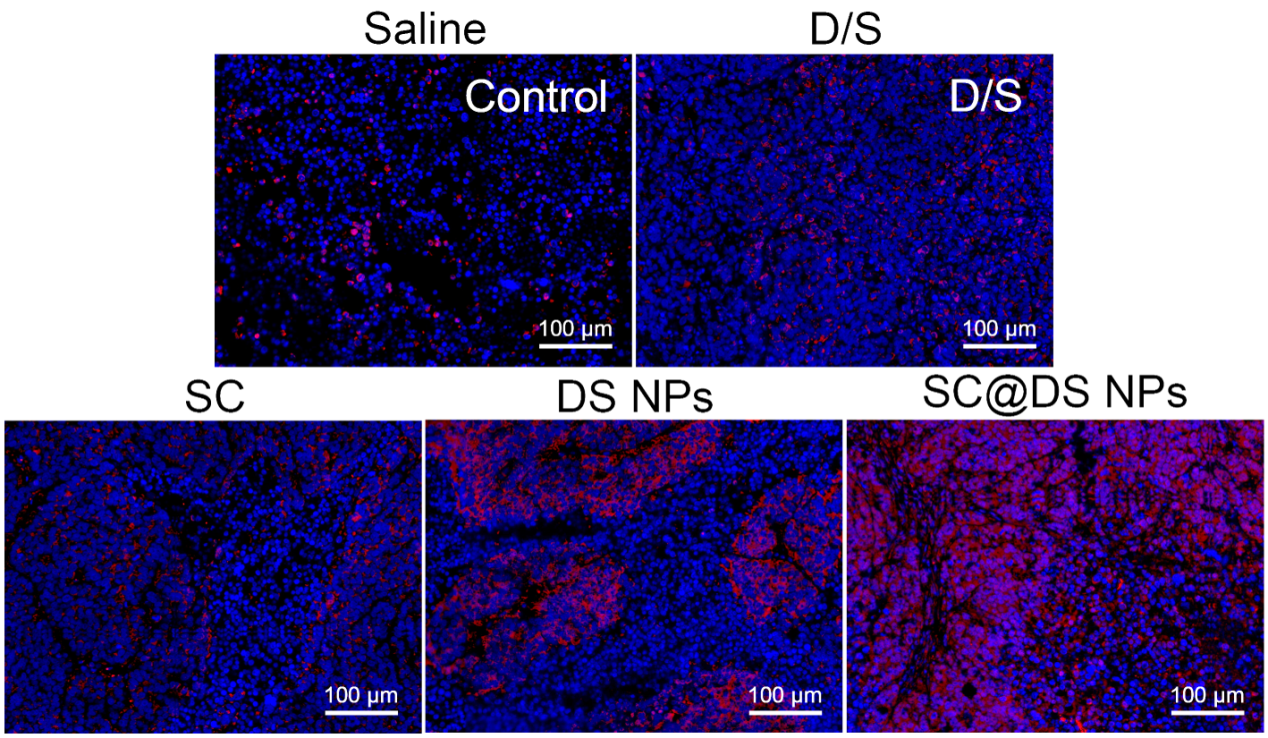


1. The protein expression of caspase 9 in tumor tissues by the immunofluorescence analysis.


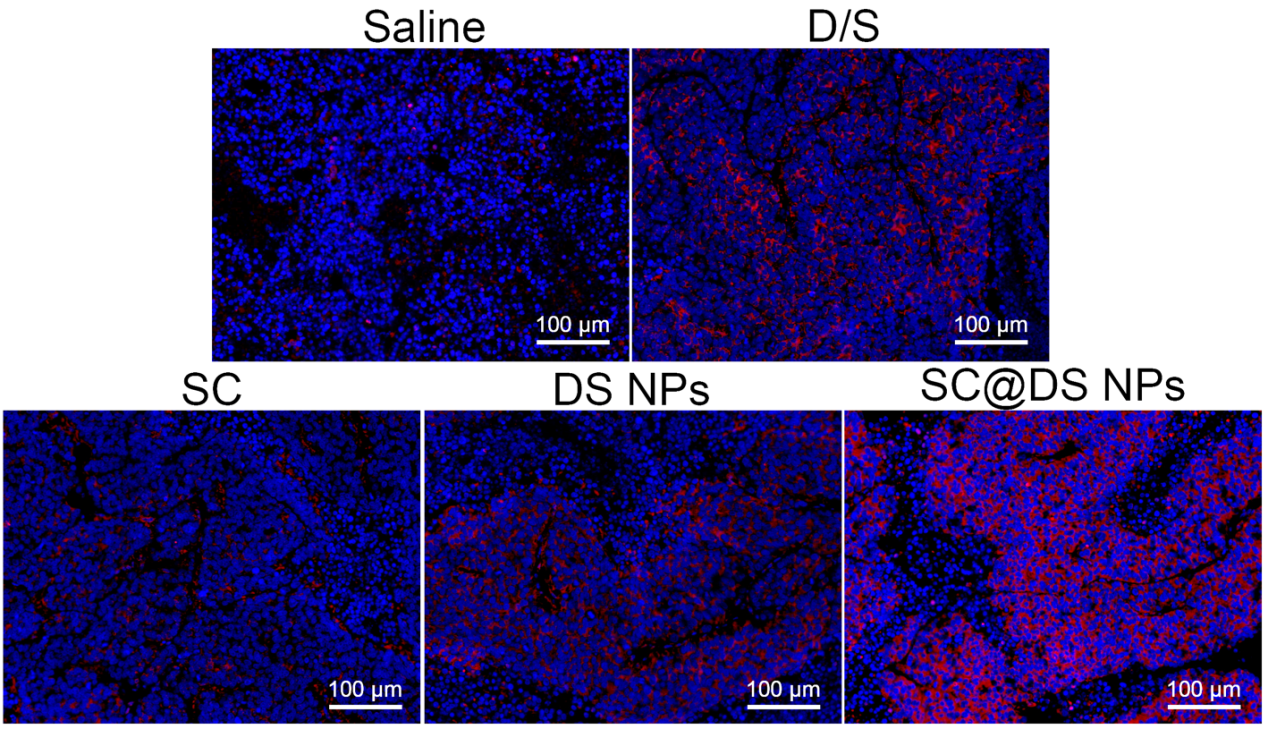


1. The protein expression of bax in tumor tissues by the immunofluorescence analysis.


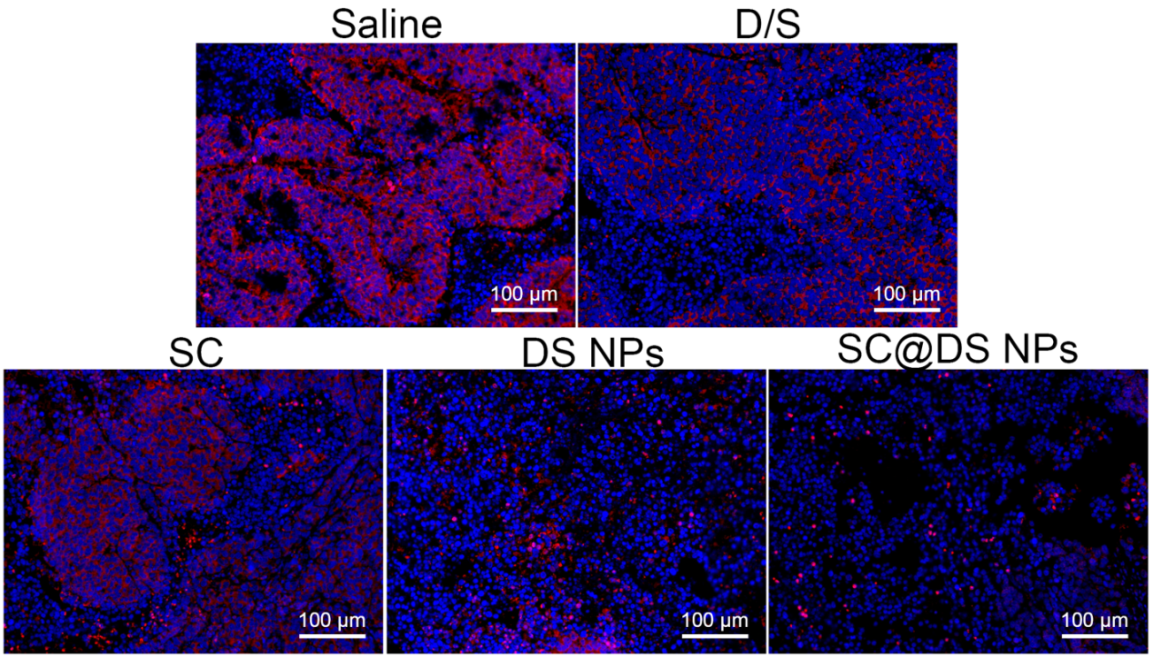


1. The protein expression of bcl-2 in tumor tissues by the immunofluorescence analysis.

Table S1 Evaluation of hydrodynamic size and PDI of SC@DS NPs after different extrusion times.


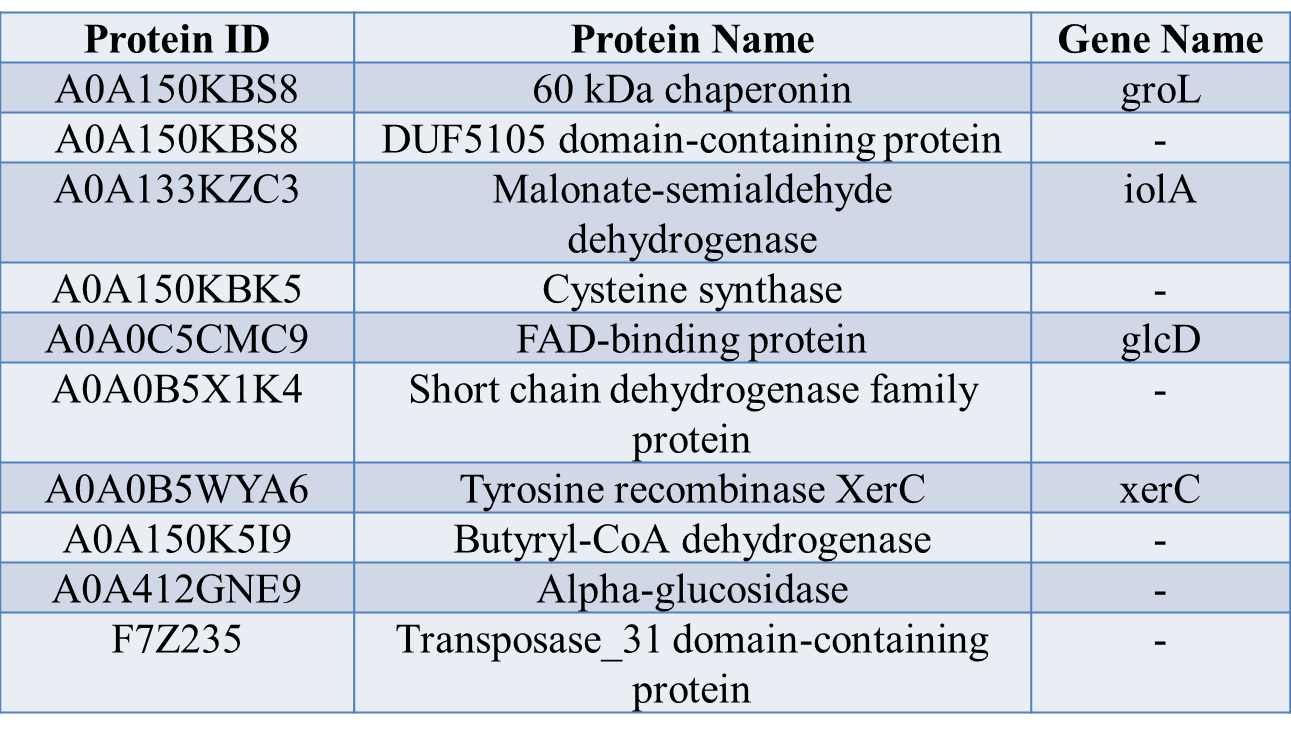


Table S2. Analysis of typical protein components of SC.


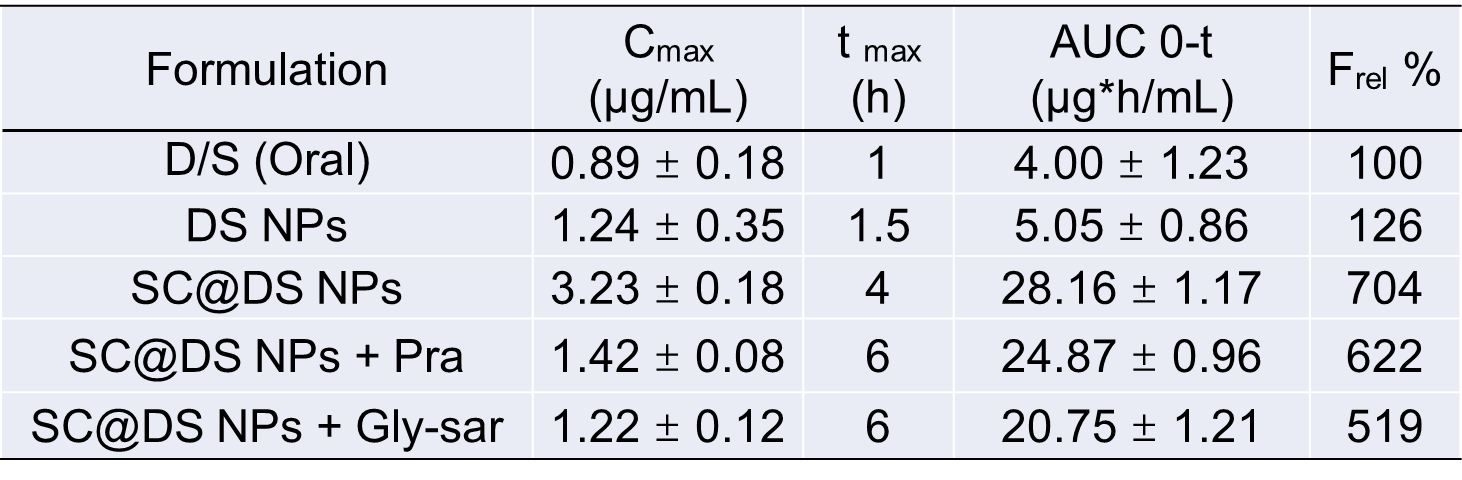


Table S3. Pharmacokinetic parameters of different samples following the oral administration.


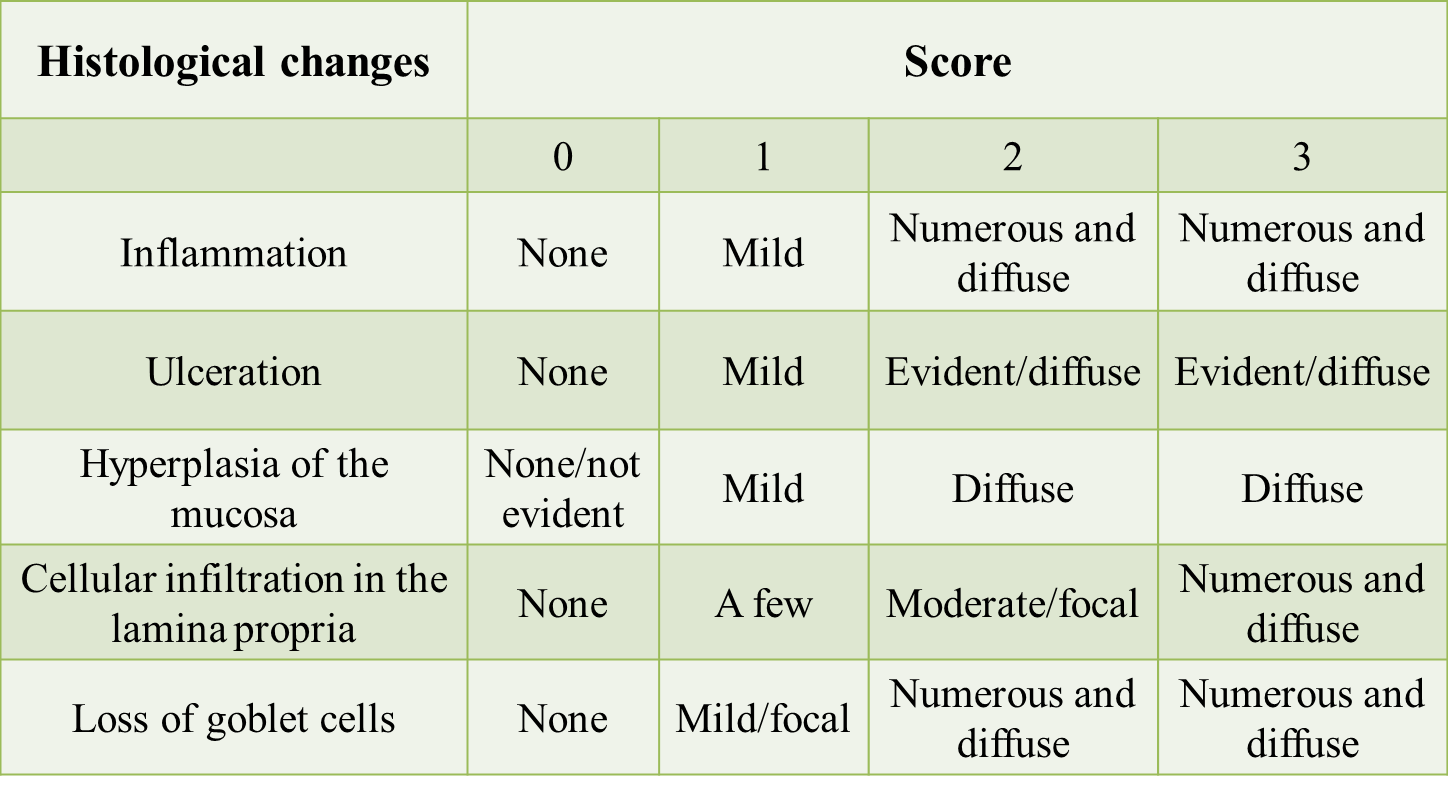


Table S4. Histological scoring parameters of different groups following the oral administration.
